# High Variability of Mitochondrial Gene Order among Fungi

**DOI:** 10.1093/gbe/evu028

**Published:** 2014-02-06

**Authors:** Gabriela Aguileta, Damien M. de Vienne, Oliver N. Ross, Michael E. Hood, Tatiana Giraud, Elsa Petit, Toni Gabaldón

**Affiliations:** ^1^Bioinformatics and Genomics Programme, Centre for Genomic Regulation (CRG), Barcelona, Spain; ^2^Universitat Pompeu Fabra (UPF), Barcelona, Spain; ^3^INRA, UR1052 GAFL, Montfavet, France; ^4^Mediterranean Centre for Marine and Environmental Research (ICM-CSIC), Barcelona, Spain; ^5^Mediterranean Institute of Oceanography (MIO) Aix-Marseille University, CNRS/INSU, IRD, Marseille, France; ^6^Biology Department, Amherst College; ^7^CNRS, UMR 8079 Ecologie, Systématique et Evolution, Orsay, France; ^8^UMR 8079 Université Paris Sud, Ecologie, Systématique et Evolution, Orsay, France; ^9^Institució Catalana de Recerca i Estudis Avancats, Barcelona, Spain; ^10^Present address: Université Lyon 1, CNRS, UMR 5558, Laboratoire de Biométrie et Biologie Evolutive

**Keywords:** Basidiomycota, sordariomycetes, basal fungi, fungal phylogeny, rearrangement rates, genome size reduction, endosymbiosis

## Abstract

From their origin as an early alpha proteobacterial endosymbiont to their current state as cellular organelles, large-scale genomic reorganization has taken place in the mitochondria of all main eukaryotic lineages. So far, most studies have focused on plant and animal mitochondrial (mt) genomes (mtDNA), but fungi provide new opportunities to study highly differentiated mtDNAs. Here, we analyzed 38 complete fungal mt genomes to investigate the evolution of mtDNA gene order among fungi. In particular, we looked for evidence of nonhomologous intrachromosomal recombination and investigated the dynamics of gene rearrangements. We investigated the effect that introns, intronic open reading frames (ORFs), and repeats may have on gene order. Additionally, we asked whether the distribution of transfer RNAs (tRNAs) evolves independently to that of mt protein-coding genes. We found that fungal mt genomes display remarkable variation between and within the major fungal phyla in terms of gene order, genome size, composition of intergenic regions, and presence of repeats, introns, and associated ORFs. Our results support previous evidence for the presence of mt recombination in all fungal phyla, a process conspicuously lacking in most Metazoa. Overall, the patterns of rearrangements may be explained by the combined influences of recombination (i.e., most likely nonhomologous and intrachromosomal), accumulated repeats, especially at intergenic regions, and to a lesser extent, mobile element dynamics.

## Introduction

Mitochondria play various essential roles in eukaryotic cells, particularly with respect to their primary functions in respiratory metabolism and energy production. From its origin as a proto-mitochondrion derived from an alpha-proteobacterium to its current state as a cellular organelle, major changes have occurred not only in terms of protein repertoire but also in the organization of the mitochondrial (mt) genome (Adams and Palmer 2003; Gabaldon and Huynen 2004). Previous studies have shown that subsequent to the endosymbiotic origin of mitochondria, a high percentage of the ancestral alpha-proteobacterial protein-coding genes were lost and replaced by proteins of different origins (Gabaldon and Huynen 2003; Gabaldon and Huynen 2007). The loss of ancestral bacterial genes resulted in significant genome size reduction (Bullerwell and Lang 2005).

mt genome evolution differs remarkably among the major groups of eukaryotes. Comprehensive reviews are available about mt genome evolution in animals (Boore 1999), plants (Levings and Brown 1989; Palmer et al. 2000; Kitazaki and Kubo 2010), protists (Gray et al. 1998; Burger et al. 2003), and fungi (Paquin et al. 1997), as well as comparison among these lineages (Burger et al. 2003; Bullerwell and Gray 2004). Animal mt genomes are generally gene rich, practically intron less, and they have a high rate of DNA sequence evolution. Gene order tends to be conserved, especially within major phyla although they can be variable between them (Boore 1999). In the last few years, however, examples from different animal groups, in particular molluscs (Boore and Brown 1994; Yamazaki et al. 1997; Boore 1999; Kurabayashi and Ueshima 2000; Rawlings et al. 2001), have challenged this view showing that rearrangements can occur within animal mt genomes (Perseke et al. 2008; Bernt and Middendorf 2011). An important feature is that animal mtDNAs typically do not engage in recombination (but see Rocha 2003; Rokas et al. 2003; Barr et al. 2005). In contrast, plant mt genomes have sequence characteristics that are associated with more frequent recombination, including large intergenic regions that can house repeated DNA sequences and a variable number of introns and their associated intronic open reading frames (ORFs) (Palmer et al. 2000). Such repetitive genomic elements contribute to the significant increase of mt genome size in some plants (e.g., in the *Silene* genus, Sloan et al. 2012). Also, plant mt genomes have experienced frequent rearrangements, particularly in vascular plants, and they have higher gene order variability relative to animal mt genomes (Palmer et al. 2000; Kitazaki and Kubo 2010; Galtier 2011; Liu et al. 2011 and references therein). Interestingly, algal mt genomes do not show many rearrangements and are thus a group of plants retaining many characteristics of the ancestral eukaryotic mitochondria (Liu et al. 2011). Plant mt genomes tend to have rates of DNA sequence evolution that are lower than in animals (Palmer et al. 2000; Kitazaki and Kubo 2010). They can also perform extensive RNA editing, although this capability is variable between plant lineages (Hecht et al. 2011; Liu et al. 2011). Other, less-well-studied eukaryotes include the phylogenetically diverse and nonmonophyletic protists, whose mt genomes can display the most variation in organizations (Burger et al. 2003; Bullerwell and Gray 2004). Protist mt genomes can be either linear or circular, have multiple linear chromosomes transcribed separately, and vary dramatically in size (Burger et al. 2003; Vlcek et al. 2011). There does not seem to be a generalized tendency in terms of rate of mt evolution or capacity to recombine among protists and they exhibit variability in terms of gene order (Gray et al. 1998; Burger et al. 2003).

Fungal mt genomes have been less studied than their animal or plant counterparts, yet they hold great potential for illuminating the evolution of organellar genomes. The most evident feature is that, although gene content is largely conserved, their relative gene order is highly variable, both between and within the major fungal phyla (Paquin et al. 1997 and references in table 1). One difference between the largest two fungal phyla, Ascomycota and Basidiomycota, is that in most ascomycetes, genes are typically encoded on the same mtDNA strand, whereas in basidiomycetes, they can be encoded on both mtDNA strands (references in table 1). Another remarkable characteristic is that, although fungi are a lineage more closely allied with animals, mtDNAs in these organisms show signals of recombination, a characteristic that is more similar to plant mtDNAs. Also similar to plants, fungal mt genomes can have large intergenic regions including sequence repeats and introns. Interestingly, fungi have mostly group I introns, whereas plant mitochondria tend to possess preferentially group II introns (Lang et al. 2007). Intron numbers are highly variable in fungal mtDNA; for instance, although the mitochondrion of the ascomycete *Podospora anserina* has 15 group I introns and 1 group II intron, that of the basidiomycete *Schizophyllum commune* shows no introns at all (Specht et al. 1992; Paquin et al. 1997). In fact, mt genome size variation can be explained in large part by differences in the number and length of introns: intron length can range from a few bp up to 5 kb (Lang et al. 2007). Such fungal introns often display autonomous proliferation in mt genomes via homing endonucleases (HEs), proteins with DNA endonuclease activity that allows them to move easily in the genome by intron transfer, and site-specific integration or “homing” (Lazowska et al. 1980; Lambowitz and Perlman 1990; Pellenz et al. 2002).

**Table 1:** List of the Species Analyzed, Accessions, and References

Species	Taxonomy^a^	GenBank Accession	Reference
*Allomyces macrogynus*	Ur	NC_001715	Paquin and Lang (1996)
*Arthroderma obtusum*	E	NC_012830	Wu et al. (2009)
*Beauveria bassiana*	S	NC_017842	Ghikas et al. (2010)
*Candida albicans*	S1	NC_018046	Bartelli et al. (2013)
*Candida glabrata*	S2	NC_004691	Koszul et al. (2003)
*Chaetomium thermophilum*	S	NC_015893	Amlacher et al. (2011)
*Cordyceps bassiana*	S	NC_013145	Ghikas et al. (2010)
*Cryptococcus neoformans*	B	NC_004336	Litter et al. (2005)
*Debaryomyces hansenii*	S1	NC_010166	Sacerdot et al. (2008)
*Dekkera bruxellensis*	S1	NC_013147	Prochazka et al. (2010)
*Fusarium oxysporum*	S	NC_017930	Pantou et al. (2008); Al-Reedy et al. (2012)
*Gibberella zeae*	S	NC_009493	Herring et al. (unpublished)
*Kluyveromyces lactis*	S2	NC_006077	Zivanovic et al. (2005)
*Lecanicillium muscarium*	S	NC_004514	Kouvelis et al. (2004)
*Metarhizium anisopliae*	S	NC_008068	Ghikas et al. (2006)
*Mycosphaerella graminicola*	D	NC_010222	Torriani et al. (2008)
*Microsporum canis*	E	NC_012832	Wu et al. (2009)
*Millerozyma farinosa*	S1	NC_013255	Jung et al. (2010)
*Moniliophthora perniciosa*	B	NC_005927	Formighieri et al. (2008)
*Microbotryum violaceum-Sl*	B	NC_020353	Lang (unpublished)
*Nakaseomyces bacillisporus*	S2	NC_012621	Bouchier et al. (2009)
*Ogataea angusta*	S1	NC_014805	Eldarov et al. (2011)
*Phakopsora pachyrhizi*	B	NC_014344	Stone et al. (2010)
*Paracoccidioides brasiliensis*	E	NC_007935	Cardoso et al. (2007)
*Peltigera malacea*	D	NC_016955	Xavier et al. (2012)
*Penicillium marneffei*	E	NC_005256	Woo et al. (2003)
*Phaeosphaeria nodorum*	D	NC_009746	Hane et al. (2007)
*Pichia pastoris*	S1	NC_015384	Kueberl et al. (2011)
*Pleurotus ostreatus*	B	NC_009905	Wang et al. (2008)
*Podospora anserina*	S	NC_001329	Bullerwell et al. (2000)
*Rhizophydium* sp.*136*	Ur	NC_003053	Forget et al. (2002)
*Schizosaccharomyces japonicus*	X	NC_004332	Bullerwell et al. (2003)
*Schizophyllum commune*	B	NC_003049	Forget et al. (2002)
*Tilletia indica*	B	NC_010651	Yi et al. (unpublished)
*Trametes cingulata*	B	NC_013933	Haridas and Gantt (2010)
*Ustilago maydis*	B	NC_008368	Kennell and Bohmer (unpublished)
*Vanderwaltozyma polyspora*	S2	NC_009638	Scanell et al. (unpublished)
*Yarrowia lipolytica*	X	NC_002659	Kerscher et al. (2001)

^a^Taxonomy: B, basidiomycetes; S1, saccharomycetes1; D, dothideomycetes; E, eurotiomycetes; S, sordariomycetes; Ur, early diverging or basal; X, other; S2, saccharomycetes2.

The presence of repetitive DNA within mt genomes in the form of introns and their associated traits of self-splicing and insertion endonuclease activity may contribute to the structural dynamics of fungal mt genomes, eliciting changes in gene order, overdispersal of repetitive elements, and the introduction of new genes through horizontal gene transfer (HGT) (Vaughn et al. 1995; Ferandon et al. 2010). Moreover, the repeats accumulated in mt introns have been associated with increased recombination and deletions (Rocha 2006), a process that is frequently invoked to explain differences in mt gene order in fungi but that is remarkably absent in Metazoa (Saccone et al. 2002). Finally, another factor potentially contributing to gene order variation is the distribution of transfer RNAs (tRNAs), which display editing, excision, and integration capabilities, that allow them to change location within the genome and participate in HGT events (Tuller et al. 2011). Because changes in tRNA location are relatively rare events, tRNA location within fungal mt genomes has been used to study fungal evolution and phylogenetic signal (Cedergren and Lang 1985).

To date, a number of studies have provided insights into fungal mt genomes (see references in table 1); however, to our knowledge, there has not been a large-scale comparative analysis providing a broader picture of the evolution of fungal mt genomes, especially of the remarkable variability in gene order. Here, we therefore set out to investigate variation in gene order among fungal mt genomes, including basidiomycetes, ascomycetes, and fungi from early diverging lineages. Our species sampling provided the taxonomic depth and balance and established the context for a comprehensive analysis of gene order evolution in fungi. Basal fungal taxa, being highly divergent with respect to our sampled ascomycetes and basidiomycetes, were analyzed separately to obtain reliable alignments. We investigated possible causes of gene order variability, specifically, we assessed 1) evidence of recombination and the dynamics of gene rearrangements and 2) the role played by intergenic regions and their associated repeats, the number of introns, intronic ORFs, and tRNA distribution. Finally, we discuss how the combined roles of recombination, chromosomal rearrangements, insertion of introns, and associated mobile elements can contribute to the high variability of gene order and tRNA distribution among fungal mitochondria.

## Materials and Methods

### Data Sets

To study the evolution of gene order in a representative data set of the major fungal group, the dikarya (constituted by the basidiomycetes and the ascomycetes), we obtained the complete mt genomes and proteomes of 38 species available in GenBank (table 1). The complete list of species analyzed in our main data set (hereafter referred to as the dikarya data set) includes nine basidiomycetes: *Tilletia indica* (NC_010651), *Phakopsora pachyrhizi* (NC_014344), *Pleurotus ostreatus* (NC_009905), *Cryptococcus neoformans* (NC_004336), *Microbotryum lychnidis-dioicae* (NC_020353), *Moniliophthora perniciosa* (NC_005927), *S. commune* (NC_003049), *Trametes cingulata* (NC_013933), *Ustilago maydis* (NC_008368); 27 ascomycetes: *Arthroderma obtusum* (NC_012830), *Beauveria bassiana* (NC_017842), *Cordyceps bassiana* (NC_013145), *Candida albicans* (NC_018046), *Candida glabrata* (NC_004691), *Chaetomium thermophilum* (NC_015893), *Debaryomyces hansenii* (NC_010166), *Dekkera bruxellensis* (NC_013147), *Fusarium oxysporum* (NC_017930), *Gibberella zeae* (NC_009493), *Kluyveromyces lactis* (NC_006077), *Lecanicillium muscarium* (NC_004514), *Metarhizium anisopliae* (NC_008068), *Microsporum canis* (NC_012832), *Millerozyma farinosa* (NC_013255), *Mycosphaerella graminicola* (NC_010222), *Nakaseomyces bacillisporus* (NC_012621), *Ogataea angusta* (NC_014805), *Paracoccidioides brasiliensis* (NC_007935), *Peltigera malacea* (NC_016955), *Penicillium marneffei* (*Talaromyces marneffei*) (NC_005256), *Phaeospheria nodorum* (*Stagonospora nodorum*) (NC_009746), *Pichia pastoris* (NC_015384), *P. anserina* (NC_001329), *Schizosaccharomyces japonicus* (NC_004332), *Vanderwaltozyma polyspora* (NC_009638) and *Yarrowia lipolytica* (NC_002659); and 2 early diverging fungi as outgroups, *Allomyces macrogynus* (NC_001715) and *Rhizophydium* sp. 136 (NC_003053).

The basal fungi data set included representatives of the main basal clades: 1) Blastocladiomycota: *Al. macrogynus* (used as outgroup in the dikarya data set: NC_001715), *Blastocladiella emersonii* (NC_011360); 2) Chytridiomycota: *Rhizophydium* sp. (used as outgroup in the dikarya data set: NC_003053); 3) Cryptomycota: *Rozella allomycis* (NC_021611); 4) Glomeromycota: *Gigaspora margarita* (NC_016684), *Glomus intraradices* (NC_012056); and 5) Monoblepharidomycota: *Harpochytrium* sp. *JEL105* (NC_004623), *Hyaloraphydium curvatum* (NC_003048), and *Monoblepharella* sp. *JEL15* (NC_004624).

### Phylogenetic Inference

To analyze the evolution of gene order through time and across the sampled species, we first reconstructed a phylogenetic tree to map the different gene orders in an evolutionary context. The two data sets defined in this study, the dikarya and the basal fungi data sets, were analyzed independently using the same methods. First, protein sequences of the orthologs shared by all sampled species, including protein-coding genes *cox*1, *cox2*, cox3, *atp*6, *atp*8, *atp*9, *nad*1, *nad*2 *nad*3, *nad*4, *nad*5, *nad*4L, and *nad*6, were aligned using a combination of six different alignment strategies (Muscle, Mafft, and dialignTX, in forward and reverse). These alignments were automatically trimmed with trimAl (Capella-Gutierrez et al. 2009) to remove poorly aligned regions based on the fraction of gaps (0.1) and the consistency across aligners (>0.16) before they were concatenated. Subsequent phylogenetic reconstruction combined neighbor joining and maximum likelihood (ML), using PhyML (Guindon et al. 2009) and RAxML v.7.2.6 (Stamatakis 2006). For the ML analyses, four substitution rate categories were used, estimating the gamma parameter and the fraction of invariable sites from the data. Support values were computed using an approximate likelihood ratio test. Bootstrap analysis was conducted with 100 resampling iterations. Once we inferred the tree, we estimated evolutionary rates with the r8s software v. 1.8 (Sanderson 2003). We used the global Langley and Fitch (LF) model, which estimates a single evolutionary rate across the whole tree (i.e., assuming a molecular clock), and the local LF models allowing for the estimation of local rates for groups of clades within the tree. The approximate ages for internal nodes were obtained using the divergence of basidiomycetes and ascomycetes (Taylor and Berbee 2006; Lucking et al. 2009), conservatively set at 500 Ma, and the whole-genome duplication event within the *Saccharomyces* clade (Wolfe and Shields 1997), set at 100 Ma. These two dates were used as calibration points. The evolutionary rates and estimated node ages were subsequently used to infer an approximate rate of rearrangements per clade.

### Whole-Genome Alignments, Recombination, and Rearrangement Events

Because whole-genome alignment methods produce better results, the more similar the genomes are, we decided to align groups of mt genomes that are not too distant in terms of sequence identity. To identify which genomes could be aligned together, we built a composite likelihood distance matrix based on the concatenated alignment of protein-coding genes *cox*1, *cox*2, *cox*3, *atp*6, *atp*8, and *atp*9. We determined the Euclidian phylogenetic distance and used the hierarchical agglomerative clustering method available in R (R Development Core Team 2011), with *h* = 0.4 to determine the groups of most closely related genomes that could be used for whole-genome alignment. With the dikarya data set, we obtained the nine following groups (hereafter referred to as fungal clusters): 1) “basidios1,” including *Tr. cingulata*, *Mo. perniciosa*, *S. commune*, and *Pl. ostreatus*; 2) “basidios2,” including *T. indica*, *U. maydis*, and *C. neoformans*; 3) “basidios3,” including *M. violaceum-Sl* and *Ph. pachyrhizi*; 4) “sordariomycetes,” including *B. bassiana*, *Co. bassiana*, *Ch. thermophilum*, *P. anserina*, *F. oxysporum*, *G. zeae*, *L. muscarium*, and *Me. anisopliae*; 5) “saccharomycetes1,” including *Ca. albicans*, *D. bruxellensis*, *De. hansenii*, *Mil. farinosa*, *O. angusta*, and *Pi. pastoris*; 6) “saccharomycetes2,” including *Ca. glabrata*, *K. lactis*, *N. bacillisporus*, and *V. polyspora*; 7) “dothideomycetes,” including *My. graminicola*, *Pel. membranacea*, and *Ph. nodorum*; 8) “eurotiomycetes,” including *A. obtusum*, *Mi. canis*, *Pa. brasiliensis*, and *Pen. marneffei*; and 9) “basals,” including *Al. macrogynus* and *Rhizophydium* sp. Neither *Schizos. japonicus* nor *Y. lipolytica* could be reliably aligned with the other clusters so they were excluded from further analysis. The complete mt genomes in each cluster were aligned with Mauve v.2.3.1 (Darling et al. 2010) using the full alignment option. This general-purpose multiple sequence aligner is able to handle whole-genome alignments and has the advantage that it identifies syntenic blocks despite rearrangements and reversals. We further refined the alignments of the syntenic blocks using t-coffee (Notredame et al. 2000) and analyzed them with GRIMM v. 1.04 (Tesler 2002) and UniMoG (Hilker et al. 2012) to infer a minimal history of rearrangements among the aligned genomes. We assumed the Double-Cut and Join (DCJ), restricted DCJ, Hannenhalli and Pevzner (HP), inversion, and translocation models. These methods predict optimized rearrangement scenarios between pairs of gene order lists. GRIMM infers inversions and takes only lists of gene orders including the same number of genes, in other words, it does not take into account gene losses, whereas UniMoG does. The latter has the advantage that it extends the DCJ to include the HP, inversion, and translocation models. Finally, the syntenic blocks, the intergenic regions, and the individual one-to-one orthologs of all genomes were tested for recombination, the most likely mechanism explaining the observed gene order variability.

There are several methods available to detect different types and signals of recombination (Martin et al. 2011). In our case, we needed methods that could identify incongruent blocks of sequence along the alignments. We chose methods that look for incongruence in terms of patterns of sites, including RDP3 v.4.16 (Martin et al. 2010), PhiPack (Bruen et al. 2006), and Recco (Maydt and Lengauer 2006). However, as far as we know, there is no specific software for the detection of nonhomologous intrachromosomal recombination, so it is possible that the methods available do not perform optimally for identifying this type of event. Nevertheless, looking for evidence of intrachromosomal recombination is often coincident with identifying breakpoints, reversals, and translocations, so the rearrangements we inferred using GRIMM (Tesler 2002) and UniMoG (Hilker et al. 2012) were used as a proxy for the particular case of intrachromosomal recombination.

### Gene Order Variability: Modeling Gene Order Evolution

Gene order can be studied directly by the comparison of the sequential order of mt genes described in their respective articles and/or genetic maps (see references in table 1). We used these data to investigate the most likely evolutionary scenarios: We estimated the gene order conservation (GOC) index as described in Rocha (2006) and the branch-specific GOC described in Fischer et al. (2006). GOC is simply defined as the number of contiguous ortholog pairs that are in common between compared genomes, normalized by the number of shared orthologs. Conversely, gene order loss (GOL) is defined as 1-GOC. As described in Rocha (2006), the empirical models defined in that study attempt to fit the loss of GOC with respect to time. Model 0, proposed by Tamames (2001), is the simplest model that approximates GOC to a sigmoidal curve described by GOC = 2/1 + e^α^*^t^*, where parameter α is adjusted by regression. Model 1 fits time dependence with a square root dependence, thus GOC = 1 – √α*t*. Model 2 considers that GOC decreases with time in a negative proportion to the square of the GOC at time *t*, hence 1/GOC = α*t* + 1. Finally, Rocha (2006) proposes a probabilistic approach where the probability (*P*) of two genes staying together after *t* consecutive generations is given by *P* = *p^t^*. Thus, in Model 3: GOC = *p^t^.* Note that this expression assumes that *P* is the same for all genes, which is thus somewhat unrealistic. We decided to also use the approach described in Fischer et al. (2006), where a measure of GOL for each branch in the tree is obtained and is thus more specific than the previously described empirical models. Branch-specific GOL (bsGOL) scores are obtained by minimizing the sum, over all the possible pairwise comparisons at hand, of the squared differences between the frequency of the observed GOL events and the sum of the predicted branch-specific values. The following expression is minimized to obtain the bsGOL scores:

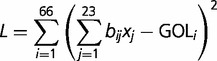

where *b_i_*_,_*_j_* is a Boolean variable that specifies the branches that are relevant for the estimation of a particular bsGOL (i.e., 0 if it is not relevant and 1 if it is), *x_j_* is obtained by minimizing *L* and is the actual bsGOL value, and GOL*_i_* are the estimated values from the pairwise comparisons, in other words, GOL*_i_* = 1 − GOC*_i_* (Fischer et al. 2006).

We approximated the GOC and bsGOL models described above to determine which of these models best fitted the data. In an attempt to better understand the process of gene order shuffling, we conducted a test to verify whether the changes observed in gene order occur randomly or whether they suggest other forces at work: Briefly, for each genome, we listed the order of genes, made all possible pairwise comparisons of these lists, estimated the GOC score (Rocha 2006), shuffled randomly the gene order, and estimated a new GOC value. We repeated this procedure 100,000 times and compared the original GOC score to the distribution of the GOCs after shuffling. We applied the Bonferroni correction for multiple testing and determined the significance (*P* values) of the comparisons. This test would indicate whether GOC between a pair of genomes is significantly different from random.

### Influence of tRNA Distribution, Intergenic, and Intronic Elements on Gene Order

To determine which genomic elements play a significant role in shaping mt gene order evolution, if any, we first obtained the number of mt protein-coding genes, introns, intronic ORFs, and repeats in all our sampled genomes. We also assessed the distribution of tRNAs, which is variable across fungal mt genomes (i.e., they can be dispersed across the genome, as in the case of *Schizos. japonicus*, or present in a few interspersed clusters, as is often the case in sordariomycetes), relative to mt protein-coding genes. The number of protein-coding genes, introns, and their associated intronic ORFs, as well as the number and location of tRNAs, were obtained from the annotations available in GenBank. Subsequently, we looked for simple repeats using RepeatMasker (Smit AFA, Hubley R, Green P. RepeatMasker Open-3.0.1996-2010; http://www.repeatmasker.org, last accessed February 18, 2014) and mreps (Kolpakov et al. 2003) for detecting tandem repeats across the whole genomes. We focused on finding repeats located within intergenic regions because we hypothesize that they may be more likely to affect gene order. Additionally, we asked whether tRNAs are significantly more clustered in genomes with high GOC (i.e., where gene order is conserved) compared with genomes with low GOC. For every taxon, we listed the mt protein-coding genes and tRNAs in order; for each ordered list, we counted the number of noncontiguous tRNAs, performed 100,000 random permutations and recounted the number of noncontiguous tRNAs each time; we compared the count in the original ordered list with the distribution obtained by the permutations; we chose a 5% threshold for the significance of tRNA clustering. Finally, we investigated the influence that the amount of introns, intronic ORFs, intergenic repeats, and the number of predicted rearrangements may have on gene order variability. To this end, we employed Pearson’s χ^2^ test, Fisher’s exact tests, and randomization tests of independence to determine the correlation between the different genomic elements (i.e., number of introns, intronic ORFs, and repeats) and the number of rearrangement events predicted per fungal cluster.

## Results

Our sampling in the dikarya data set provided the necessary taxonomic context and depth for a comprehensive analysis of gene order evolution in a phylogenetic context. The mtDNA of basal fungi was analyzed separately to obtain reliable alignments.

### Phylogenetic Analysis in the Dikarya

All the 38 species analyzed in the dikarya data set have the standard core set of mt protein-coding genes (*atp*6, *atp*8, *atp*9, *cox*1, *cox*2, *cox*3, *nad*1, *nad*2, *nad*3, *nad*4, *nad*4L, *nad*5, *nad*6, *cob*, *rnl*, and a variable number of tRNAs). In addition to these genes, we found the atypical presence of *rsp3* (encoding the ribosomal protein S3) in *S. commune*, *Mo. perniciosa*, *Pl. ostreatus*, *Tr. cingulata*, and *M. lychnidis-dioicae*. We also found *rnpB* (encoding the RNA subunit of mt RNase P) in the mt genomes of *M. lychnidis-dioicae*, *S. commune*, and *U. maydis*. To our knowledge, *rnpB* has not been described in other basidiomycete mt genomes; it has so far only been recognized in mtDNAs of a few zygomycete and ascomycete fungi, two protists, and never in animals and plants (Seif et al. 2003, 2005).

**Fig. 1.—:**
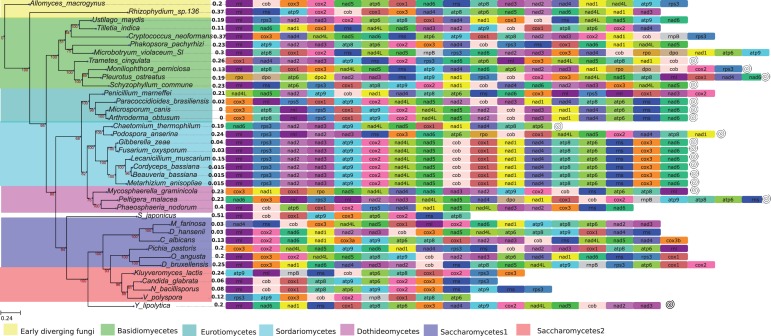
ML phylogeny of our sampled taxa including the 38 species in the dikarya data set. The gene tree was inferred from a concatenated alignment of 14 single-copy orthologous genes (*atp*6, *atp*8, *atp*9, *nad*1–*nad*6, *nad*4L, *cob*, and *cox*1–*cox*3). RAxML v.7.2.6 (Stamatakis 2006) was used assuming the LG substitution matrix and default parameters. On the right side of each taxon name is a series of colored boxes representing the mt gene order according to GenBank annotation. Bootstrap support appears next to each node. bsGOL values are shown next to each species name, and they are estimated by minimizing the following expression: *L* = ∑(∑*b_i_*_,_*_j_x_j_* – GOL*_i_*)^2^, where *b_i_*_,_*_j_* is a Boolean variable that specifies the branches that are relevant for the estimation of a particular bsGOL (i.e., 0 if it is not relevant and 1 if it is), *x_j_* is obtained by minimizing *L* and is the actual bsGOL value, and GOL*_i_* are the estimatedvalues from the pairwise comparisons, in other words, GOL*_i_* = 1 − GOC*_i_* (see Fischer et al. 2006 for more details). Significant tRNA clustering was found in species marked with a spiral. This figure was made using the ETE python environment for tree exploration (Huerta-Cepas et al. 2010).

The inferred phylogenetic tree including all 38 species in the dikarya data set (fig. 1) is in agreement with previously published fungal phylogenies (Marcet-Houben and Gabaldon 2009; Ebersberger et al. 2012), and most internal nodes are well supported (i.e., >90%). The global LF model that estimates a single evolutionary rate across the whole tree, that is, assuming a molecular clock, predicted 1.65 × 10^−^^3^ substitutions per site per time unit (Myr) and the local LF model, that is, without assuming a molecular clock, estimated 1.51 × 10^−^^3^ for the basal group (*Al. macrogynus* and *Rhizophydium* sp. *136*), 2.1 × 10^−^^3^ for the ascomycetes, and 1.35 × 10^−^^3^ for the basidiomycetes, suggesting a faster evolutionary rate in ascomycetes relative to the basidiomycetes and the basal fungi sampled in this study. This rate is lower than the reported average rates for mammals (i.e., about 33.88 × 10^−^^9^/Y, that is approximately 3.4 × 10^−^^2^/Myr; Nabholz et al. 2009) but higher than that of plant mt genomes (i.e., 0.34 × 10^−^^9^/Y, that is 3.4 × 10^−^^4^/Myr; Wolfe et al. 1987). These are only approximate comparisons, as we did not analyze population data.

### Rearrangements and Recombination in the Dikarya

Despite the overall conservation of the standard set of mt genes, we found striking variation in gene order among fungal species in the dikarya data set. Noteworthy exceptions to this trend are the *nad*4L/*nad*5 and *nad*2/*nad*3 genes, which tend to be next to each other in most species. The overlap of the stop and initiation codons between the particular genes in these two pairs is the most likely cause for their contiguity, as it occurs in *Pleurotus* mtDNA (Wang et al. 2008).

Because nonhomologous, intrachromosomal recombination is known to cause chromosomal rearrangements (Gordenin et al. 1993; Bi and Liu 1996; Lobachev et al. 1998; Rocha et al. 1999; Waldman et al. 1999; Rocha 2003, 2006; Phadnis et al. 2005; Odahara et al. 2009; Lavrov 2010), it could potentially explain the high gene order variability we observe in fungal mitochondria. We thus set out to detect recombination among the syntenic regions and whole-genome alignments in all the fungal clusters. We did find signals of recombination in most alignments but not unequivocal evidence of nonhomologous, intrachromosomal recombination, as other types of processes may have generated similar signals. To be conservative, we decided to keep only those results supported with high confidence (*P* value < 0.05), but in general, most signals did not have a high support. The average recombination rate was estimated as the number of predicted recombination events normalized by the average substitution rate obtained from r8s for each clade, per fungal cluster. The recombination events per site per time unit (Myr) were basidiomycetes (all 3 basidiomycete clusters): 0.11; sordariomycetes: 0.26; saccharomycetes1: 0.02; eurotiomycetes: 0.09; dothideomycetes: 0.06; and saccharomycetes2: 0.15. Recombination was not detected for the basal fungal cluster with high confidence. It is noteworthy that most recombination detection programs lack power when looking for sporadic traces of recombination, as it is the case in mt genomes (Posada and Crandall 2001; Barr et al. 2005; Neiman and Taylor 2009).

Arguably, a better approach for investigating the evolution of gene order due to nonhomologous, intrachromosomal recombination is to use estimates of gene rearrangements as a proxy, as both involve identifying breakpoints, inversions and translocations. We, therefore, compared the gene order lists to infer the rearrangements that occurred between all pairs of species within each of the fungal clusters in the dikarya data set. The average minimal rearrangement rates, estimated as the number of predicted rearrangement events normalized by the average substitution rate for each clade (per fungal cluster) were: 0.03 for basidiomycetes (all three basidiomycete clusters); 0.01 for sordariomycetes; 0.04 for saccharomycetes1; 0.02 for eurotiomycetes; 0.05 for dothideomycetes; 0.02 for saccharomycetes2; and 0.03 for basals. These results are consistent with the overall higher gene order variability observed in basidiomycetes, saccharomycetes2, followed by the saccharomycetes1, and in contrast to what is observed in sordariomycetes, dothideomycetes, and eurotiomycetes.

### Gene Order Variability in the Dikarya

**Fig. 2.—:**
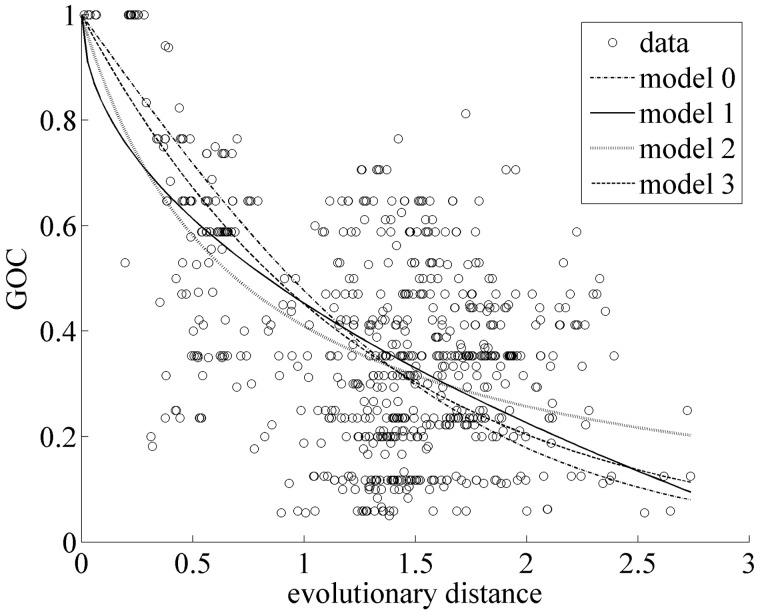
GOC between pairs of genomes of the dikarya data set as a function of their phylogenetic (patristic) distance. Distances were estimated using the estimated branch lengths in figure 3, listed in table 2. Models are fitted by nonlinear regression. Model 0: GOC = 2/1+e^α^*^t^*. Model 1: GOC = 1 – √α*t*. Model 2: 1/GOC = α*t* + 1. Model 3: GOC = p*^t^*, where parameter α is adjusted by regression and *t* is the patristic distance between the two compared taxa.

**Fig. 3.—:**
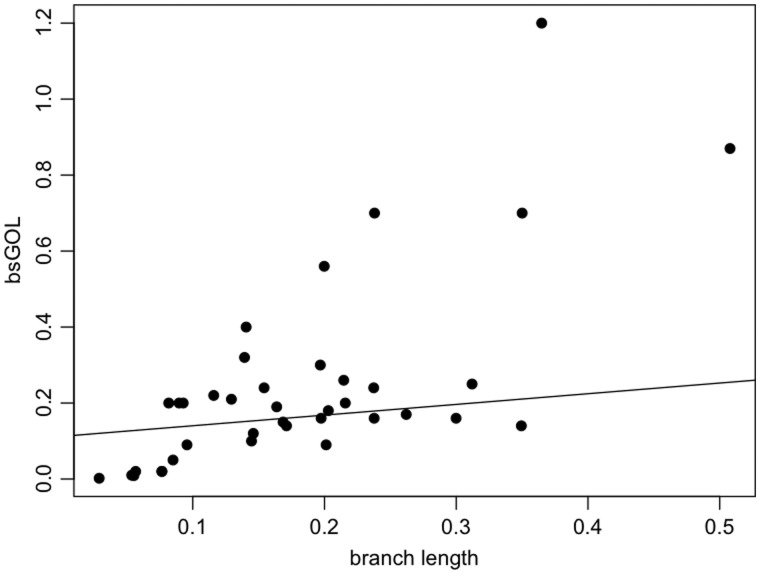
Pearson’s correlation between bsGOC values and branch lengths (*R* = 0.7, *P* value < 0.0005) for the dikarya data set.

In the dikarya data set, GOC and bsGOL scores, estimated by the methods of Rocha (2006) and Fischer et al. (2006), did not exhibit good fits to the patristic (phylogenetic) pairwise distance with tested empirical models (supplementary table S1, Supplementary Material online; fig. 2). The goodness-of-fit scores obtained were Model 0 = 27.25, Model 1 = 23.25, Model 2 = 21.2, and Model 3 = 24.43. Following Fischer’s approach (Fischer et al. 2006) to refine the models with estimates of bsGOL, gene order scores were observed to vary slightly among fungal clusters (table 2; bsGOL values on the right side of each species name in fig. 1). Nevertheless, the average bsGOL score per group captures the GOL trend differences among fungal clusters: the highest average GOL score (i.e., where GOL is greatest) is for the basidiomycetes, with 0.21, followed by the early diverging fungi (*Al. macrogynus* and *Rhizophydium* sp. 136) and the saccharomycetes2 (*K. lactis*, *Ca. glabrata*, *N. bacillisporus*, and *V. polyspora*) both at 0.2; at an intermediate average bsGOL level are the saccharomycetes1 (*Mil. farinosa*, *De. hansenii*, *Ca. albicans*, *Pi. pastoris*, *O. angusta*, and *D. bruxellensis*) at 0.18 and the dothiodeomycetes (*My. graminicola*, *Pel. malacea*, and *Ph. nodorum*) at 0.16; at the lowest level of GOL are the eurotiomycetes (*A. obtusum*, *Mi. canis*, *Pa. brasiliensis*, and *Pen. marneffei*) at 0.14 and the sordariomycetes (*C. globosum*, *P. anserina*, *G. zeae*, *F. oxysporum*, *L. muscarium*, *Co. bassiana*, *B. bassiana*, and *Me. anisopliae*) at 0.1. Also, bsGOL values show a moderate correlation with branch length values (*R* = 0.7, *P* value < 0.0005, fig. 3). This is also consistent with older clades, with deeper ancestral nodes, having more rearranged genes (e.g., basidiomycetes have a higher average bsGOL value than sordariomycetes).

**Table 2:** bsGOL, NRPS Rates, and Summary of the Number of Different Genomic Elements per Species

Taxon	bsGOL^a^	Branch Lengths^b^	GOL Rate^c^	Normalized GOL Rate^d^	NPRS Rates^e^	tRNAs^f^	Intronic ORFs^g^	Introns^h^	Repeats^i^	Intergenic Repeats^j^	Genome Size^k^
*Cryptococcus neoformans*	3.50E-01	0.7	1.05E+00	2.96E+00	0.002	20	1	2	128	9	24,874
*Moniliophthora perniciosa*	1.16E-01	0.22	3.36E-01	7.89E-01	0.001	30	11	13	1,648	272	109,103
*Microbotryum violaceum-Sl*	3.12E-01	0.25	5.62E-01	1.32E+00	0.0007	20	33	22	1,003	380	107,808
*Phakopsora pachyrhizi*	2.38E-01	0.7	9.38E-01	2.11E+00	0.002	24	2	5	1,898	31	31,825
*Pleurotus ostreatus*	1.69E-01	0.15	3.19E-01	6.38E-01	0.0009	24	9	12	645	3	73,242
*Schizophyllum commune*	1.39E-01	0.32	4.59E-01	9.09E-01	0.002	27	0	0	1,718	61	49,704
*Tilletia indica*	8.97E-02	0.2	2.90E-01	5.74E-01	0.0008	24	0	0	568	52	65,147
*Trametes cingulata*	3.00E-01	0.16	4.60E-01	9.11E-01	0.0006	25	26	26	1,196	143	91,500
*Ustilago maydis*	1.71E-01	0.14	3.11E-01	6.16E-01	0.0005	23	11	12	337	37	56,814
*Candida albicans*	2.37E-01	0.24	4.77E-01	1.22E+00	0.003	30	0	2	279	40	40,420
*Debaryomyces hansenii*	1.64E-01	0.19	3.54E-01	8.64E-01	0.004	25	4	4	389	20	29,462
*Dekkera bruxellensis*	2.15E-01	0.26	4.75E-01	1.16E+00	0.003	25	3	5	3,968	939	76,453
*Millerozyma farinosa*	2.16E-01	0.2	4.16E-01	9.83E-01	0.004	25	11	14	710	31	39,107
*Ogataea angusta*	1.41E-01	0.4	5.41E-01	1.54E+00	0.004	26	6	6	944	46	41,719
*Pichia pastoris*	1.29E-01	0.21	3.39E-01	8.76E-01	0.002	25	3	6	1,080	21	35,683
*Mycosphaerella graminicola*	1.97E-01	0.16	3.57E-01	8.43E-01	0.0007	27	0	0	218	66	43,964
*Peltigera malacea*	9.28E-02	0.2	2.93E-01	8.35E-01	0.001	26	17	20	468	31	62,785
*Phaeosphaeria nodorum*	2.03E-01	0.18	3.83E-01	1.09E+00	0.0009	27	2	4	667	36	49,761
*Arthroderma obtusum*	7.66E-02	0.02	9.66E-02	2.48E-01	0.0007	25	1	1	325	14	24,105
*Microsporum canis*	7.66E-02	0.02	9.66E-02	2.38E-01	0.0007	25	1	1	344	10	23,943
*Paracoccidioides brasiliensis*	1.54E-01	0.24	3.94E-01	9.26E-01	0.001	25	3	13	552	175	71,335
*Penicillium marneffei*	2.38E-01	0.16	3.98E-01	9.19E-01	0.0007	28	9	11	303	16	35,438
*Beauveria bassiana*	2.90E-02	0.002	3.10E-02	7.38E-02	0.0002	25	5	5	284	10	29,961
*Chaetomium thermophilum*	1.45E-01	0.1	2.45E-01	6.29E-01	0.0008	28	3	3	927	113	127,206
*Cordyceps bassiana*	5.53E-02	0.009	6.43E-02	1.65E-01	0.0005	24	4	5	295	10	32,263
*Fusarium oxysporum*	5.37E-02	0.01	6.37E-02	1.66E-01	0.0006	25	1	2	298	1	34,477
*Gibberella zeae*	5.67E-02	0.02	7.67E-02	2.00E-01	0.0006	28	33	35	529	48	95,676
*Lecanicillium muscarium*	8.50E-02	0.05	1.35E-01	3.54E-01	0.002	25	1	1	241	9	24,499
*Metarhizium anisopliae*	1.46E-01	0.12	2.66E-01	6.97E-01	0.001	24	1	1	220	2	24,673
*Podospora anserina*	2.01E-01	0.09	2.91E-01	6.87E-01	0.0008	27	31	34	326	48	100,314
*Allomyces macrogynus*	1.97E-01	0.3	4.97E-01	7.44E-01	0.0004	25	10	28	219	14	57,473
*Rhizophydium* sp.*136*	3.65E-01	1.2	1.56E+00	3.84E+00	0.002	7	1	1	809	13	68,834
*Schizosaccharomyces japonicus*	5.08E-01	0.87	1.38E+00	3.38E+00	0.002	25	3	2	928	94	80,059
*Yarrowia lipolytica*	2.00E-01	0.56	7.60E-01	1.76E+00	0.002	27	15	17	792	72	47,916
*Candida glabrata*	9.56E-02	0.09	1.86E-01	2.78E-01	0.001	23	3	3	797	68	20,063
*Kluyveromyces lactis*	3.49E-01	0.14	4.89E-01	1.09E+00	0.001	22	3	3	1,190	279	40,291
*Nakaseomyces bacillisporus*	8.18E-02	0.2	2.82E-01	7.33E-01	0.003	23	0	0	7,896	3,791	107,123
*Vanderwaltozyma polyspora*	2.62E-01	0.17	4.32E-01	1.06E+00	0.002	23	0	0	1,258	186	21,684

^a^bsGOL values were estimated by minimizing the following expression: *L* = ∑(∑*b_i_*_,_*_j_x_j_* – GOL*_i_*)^2^, where *b_i_*_,_*_j_* is a Boolean variable that specifies the branches that are relevant for the estimation of a particular bsGOL (i.e., 0 if it is not relevant and 1 if it is), *x_j_* is obtained by minimizing *L* and is the actual bsGOL value, and GOL*_i_* are the estimated values from the pairwise comparisons, in other words, GOL*_i_* = 1 − GOC*_i_* (see Fischer et al. [2006] for more details).

^b^These branch lengths were obtained by ML phylogenetic reconstruction with RaxML (Stamatakis 2006).

^c^bsGOL normalized by the branch length.

^d^bsGOL rates normalized relative to the mean GOL rate value.

^e^Rates obtained with r8s with the nonparametric method minimizing local transformations (NPRS) and optimization via Powell’s method (Sanderson 2003).

^f^Number of tRNAs (GenBank).

^g^Number of intronic ORFs (GenBank).

^h^Number of introns (GenBank).

^i^Number of repeats (whole genome) detected with Repeatmasker (Smit AFA, Hubley R, Green P. RepeatMasker Open-3.0.1996-2010; http://www.repeatmasker.org, last accessed February 18, 2014).

^j^Number of intergenic repeats detected with mreps (Kolpakov et al. 2003).

^k^Genome size in bp (GenBank).

### Influence of tRNA Distribution, Intergenic, and Intronic Elements on Gene Order in the Dikarya

Rearrangements of the fungal mt genomes were influenced by the sequence characteristics, in particular the amount of repetitive DNA elements at intergenic regions. The average bsGOL value, normalized by the number of fungal species in each cluster, did not display significant correlation with any of the numbers of genomic elements (i.e., with either the number of repeats, the number of introns and their associated intronic ORFs, or the number and location of tRNAs, data not shown). Also, correlations between rearrangements and the proportions of introns and intronic ORFs were not significant (supplementary table S2, Supplementary Material online). However, the number of rearrangement events was significantly correlated with the proportion of repeats at intergenic regions (table 3): Pearson’s χ^2^ test (observed χ^2^ = 1,158.37, df = 5, *P* value < 0.0001, alpha = 0.05), Fisher’s exact tests (*P* value two-tailed < 0.0001, alpha = 0.05), Wilk’sG2 test of independence (observed Wilk’s G2 value = 1,156.383, df = 5, *P* value < 0.0001), and a randomization test of independence with 5,000 Monte Carlo simulations (observed χ^2^: 1,158.37, df = 5, *P* value < 0.0001, alpha = 0.05). Together, these results are consistent with the hypothesis that repeats favor recombination events, thereby promoting rearrangements that change gene order (Gordenin et al. 1993; Bi and Liu 1996; Lobachev et al. 1998; Rocha et al. 1999; Waldman et al. 1999; Rocha 2003, 2006; Phadnis et al. 2005; Odahara et al. 2009; Lavrov 2010). In general, the more intergenic repeats in fungal mt genomes, the more likely it was to observe rearrangements and, therefore, gene order variability.

**Table 3:** Number of Intergenic Repeats Normalized by the Number of Species in Each Fungal Cluster, with and without Outliers, and Rearrangement Events per Fungal Cluster

Fungal Cluster	Intergenic Repeats	Intergenic Repeats (without Outliers)^a^	Rearrangement Events
Basidiomycetes	988 (109.78)	336 (37.33)	414
Sordariomycetes	241 (30.13)	32 (4)	42
Dothideomycetes	133 (44.33)	133 (44.33)	24
Eurotiomycetes	215 (53.75)	40 (10)	22
Saccharomycetes1	1,097 (182.83)	158 (26.33)	156
Saccharomycetes2	4,324 (1,081)	254 (63.5)	22
Basals	27 (13.5)	27 (13.5)	14

^a^Outliers are defined as the species that have higher than average repeat content relative to their cluster: *Dekkera bruxellensis*, *Paracoccidioides brasiliensis*, *Microbotryum violaceum-Sl*, *Moniliophthora perniciosa*, *Chaetomium thermophilum*, *Gibberella zeae*, *Podospora anserina*, *Nakaseomyces bacillisporus*, and *Kluyveromyces lactis.*

**Fig. 4.—:**
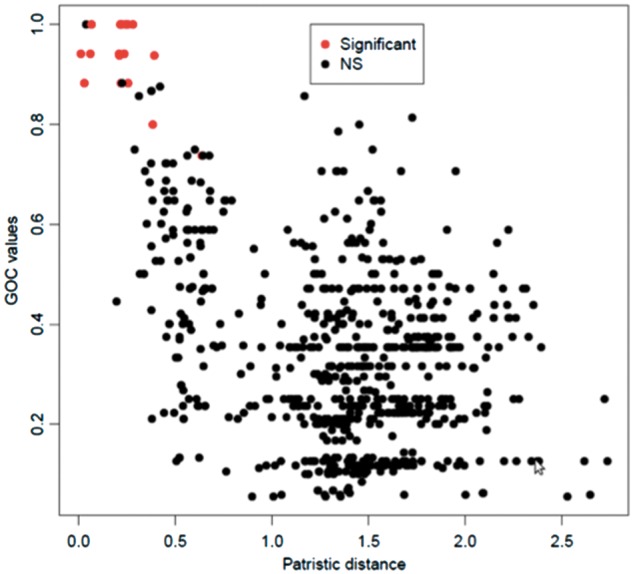
Pairwise GOC values as a function of the phylogenetic (patristic) distance between them, for the dikarya data set. Here, we conducted a randomization test as follows: for each genome, we listed the order of genes, made all possible pairwise comparisons of these lists, estimated the GOC score (Rocha 2006), shuffled randomly the gene order, and estimated a new GOC value. We obtained 100,000 reshufflings and compared the original GOC to the distribution of the shuffled GOCs. We applied the Bonferroni correction for multiple testing and determined the significance (*P* values) of the comparisons. The red dots represent significant *P* values, which correspond to the group of sordariomycetes (in fig. 1, the clade grouping *Chaetomium thermophilum*, *Podospora anserina*, *Gibberella zeae*, *F. oxysporum*, *Lecanicillium muscarium*, *Cordyceps bassiana*, *Beauveria bassiana*, and *Metarhizium anisopliae*).

The randomization test assessing pairwise GOC distributions and shuffled distributions relative to the patristic (phylogenetic) distance showed that the pairs of species whose gene order has evolved significantly differently from random correspond to the well-conserved gene order sets of the sordariomycetes (figs. 1 and 4). According to our randomization test, the other pairs of species have seen their mt DNA gene order change more or less randomly. The random permutation test implemented showed that the groups of species with highly conserved gene order, such as sordariomycetes, also showed tRNAs significantly grouped together in a few separate clusters along the mt chromosome (shown with a spiral on the right side in fig. 1). On the contrary, in species with high gene order variability (e.g., basidiomycetes), tRNAs tended to be scattered along the chromosome, consistent with the idea of tRNAs being associated to transposable elements that contribute to the variability in their distribution (Devine and Boeke 1996; Hughes and Friedman 2004) and favor the reorganization in the mt genome. Despite the presence of a few species with low gene order variability and significantly clustered tRNAs (*Y. lipolytica*, *S. commune*, *Pl. ostreatus*, *Mo. perniciosa,* and *Tr. cingulata*), we nevertheless detected a trend for most species with conserved gene order to have significantly clustered tRNAs and species with low conservation order to have more scattered tRNAs.

### Gene Order Variability in Basal Fungi

Basal fungal taxa, being highly divergent with respect to ascomycetes and basidiomycetes, were analyzed separately to obtain reliable alignments. On the basis of the similarity matrix obtained for the basal data set, we performed a clustering analysis (previously described for the dikarya data set) that resulted in three clusters of basal species that could be reliably aligned together. We thus aligned the Blastocladiomycetes: *Al. macrogynus* with *Bl. emersonii*, the Glomeromycetes: *Gi. margarita* with *Gl. intraradices*, and the Monoblepharidomycetes: *Harpochytrium* sp. together with *H. curvatum* and *Monoblepharella* sp.

No recombination events were reliably detected in any of the alignments of basal fungi. The evolutionary rates (substitutions per site per Myr) predicted by r8s assuming the NPRS model were: 8 × 10^−^^4^,1 for the Blastocladiales, 6 × 10^−^^4^ for the Monoblepharidales, and 1 × 10^−^^3^ for the Glomeromycota. The rearrangement rate per clade per Myr, as predicted by UniMoG and r8s were: 0.007 for the Blastocladiales, 0.02 for the Monoblepharidales, and 0.06 for the Glomeromycota. Supplementary figure S1, Supplementary Material online, shows the tree inferred for basal fungi, including bsGOL, branch length, and bootstrap estimates. Results are summarized in supplementary tables S3–S5, Supplementary Material online. Pairwise GOC models are shown in supplementary figure S2, Supplementary Material online. The goodness of fit scores obtained for these empirical GOC models were Model 0: 0.93, Model 1: 1.36, Model 2: 0.95, and Model 3: 0.9. bsGOL showed a moderate correlation with phylogenetic distance (*R* = 0.7, *P* value = 0.004, supplementary fig. S3, Supplementary Material online), and none of the sampled variables including introns, intronic ORFs, tRNAs or repeats appeared to have an effect on gene order. However, given the low number of data points available for the correlation analysis, we take these results with caution, as there may be low detection power. We therefore suggest that additional basal fungi need to be available before stronger conclusions can be drawn about the proximal causes of gene order variability. Overall, these results suggest that the mitochondria in basal fungi have evolved with a faster evolutionary rate relative to ascomycetes and basidiomycetes. On the other hand, mtDNA in basal fungi show comparable rates of rearrangements (an average of 0.029 events/Myr) with respect to ascomycetes and basidiomyctes, with the notable exception of Blastocladiomycetes (which exhibit a lower rate by one order of magnitude).

## Discussion

Lynch et al. (2006) have pointed out that nonselective forces such as drift and mutation may account for major differences in organelle genome structure among animals, plants, and unicellular eukaryotes. Mutation rates for mtDNA vary strikingly between these groups of organisms, with animals at the highest mutation rate spectrum and plants at the lowest. According to our results, fungal mtDNAs lie at intermediate mutation rates between animal and plant mtDNA. Important features are shared between fungal and plant mtDNA: lower substitution rates than in animal mitochondria, the presence of introns and associated mobile elements, higher noncoding DNA than in animal mt genomes, and the capability of recombination and the presence of recombination-associated DNA repair mechanisms. Galtier (2011) has suggested that life cycle, metabolic, and ageing (senescence) differences may explain these striking differences between animal and plant mtDNA. We argue that such differences could also explain the discrepancies with respect to animal mtDNA and the similarities with plant mt genomes, although these comparisons have not been specifically addressed, as far as we know.

Here, we have shown that there is high variability in terms of mt gene order among fungi, both between and within the major phyla (i.e., basidiomycetes, ascomycetes, and early diverging fungi). The mt genomes of basidiomycetes are in general among the most rearranged groups (average bsGOL = 0.21), but other groups defined in this study, in particular the saccharomycetes1 and saccharomycetes2, also show high variability (bsGOL = 0.2 and 0.18, respectively). On the contrary, the sordariomycetes and the eurotiomycetes have highly conserved gene arrangements (bsGOL = 0.1 and 0.14, respectively). Although GOL can occur rapidly within a clade, as seen with the pairwise GOC models, it does not appear to increase linearly with time. The average bsGOL scores are somewhat more powerful at detecting trends in GOL than the empirical models for pairwise GOC. This means that, even if it is not very strong, there is a phylogenetic component to gene order variability patterns. Moreover, bsGOL scores show a moderate correlation with branch lengths. This indicates that time contributes somewhat to GOL although there could be other confounding factors (e.g., gene loss in the saccharomycetes2 and *Schizos. japonicus*).

GOC/GOL models measure gene order variability through time but do not offer a mechanistic explanation of this process. We used a simple nonparametric randomization test to try to identify the propensity of particular fungal groups to have greater change in gene orders. What our test showed is that, except for the sordariomycetes, which have remarkably conserved gene order within the group, all other fungal clusters seem to have genes rearranged more or less randomly. One could think that sordariomycetes display a highly conserved gene order because they constitute a relatively young fungal group. Nevertheless, in the same time unit of a million years, they have the lowest rearrangement rate compared with the other fungal clusters. Time alone does not explain gene order changes.

Among the genomic elements studied here, repeats at intergenic regions show the strongest correlation with gene order. According to theoretical studies, the accumulation of repeats, among other mtDNA structural features, seems to be driven mostly by drift and mutation pressure, which are in turn largely determined by population size dynamics (e.g., genome size reduction or bottlenecks (Lynch and Blanchard 1998; Lynch et al. 2006). Although intron-associated ORFs, in particular those encoding HEs, have a great potential to insert copies in different locations within the genome, thereby changing gene order, we did not observe a strong correlation with gene rearrangements. If they play a role in shaping gene order it appears to be less important than that of simple and tandem repeats, especially those repeats present at intergenic regions.

The distribution of tRNAs contributes to protein-coding gene order variation among fungi, as they themselves can change location (Perseke et al. 2008). tRNAs have been associated with breakpoints involved in nuclear chromosomal rearrangements (Di Rienzi et al. 2009), and our results about fungal mtDNAs are consistent with this observation. Species showing the highest gene order variability are those showing a scattered tRNA distribution (e.g., *Schizos. japonicus*), as opposed to less variable species, whose tRNAs tended to be clustered (e.g., in sordariomycetes). Another source of gene order variability is gene loss (e.g., due to transfers to the nucleus), which could be important in the saccharomycetes2 and *Schizos. japonicus*. Finally, although less frequent, HGTs can also contribute to gene order changes (e.g., Bergthorsson et al. 2004) but we did not investigate it here.

A commonly invoked mechanism to explain gene order changes is the “tandem-duplication-random-loss” model (Lavrov et al. 2002) that was first proposed to explain gene order in millipedes and suggested that the entire mtDNA was duplicated with a subsequent loss of gene blocks. In our case, this model could partly explain the gene order differences (only the loss and not the duplication) observed among saccharomycetes2 and in *Schizos. japonicus* due to the gene loss of the NADH gene family (Gabaldon et al. 2005), as these losses necessarily resulted in gene order changes relative to the ancestral gene arrangement that included the NADH genes. The tandem-duplication-random-loss model, however, cannot account for inversions and transpositions, which may be better explained by nonhomologous, intrachromosomal recombination. We suggest that most of the observed gene rearrangements among fungal mtDNAs are very likely caused by this or a similar recombinational mechanism. Notably, recombination has been reported in vitro and in natural populations for fungal mt genomes (van Diepeningen et al. 2010).

Difficulties in detecting recombination based on sequence data can result from multiple factors, including the scale of the genomic regions involved, where analysis of adjacent nucleotides may fail to detect recombination occurring across large physical distances (Neiman and Taylor 2009) or where sequences are not divergent enough for software to detect them (at least two phylogenetically informative sites must exist to each side of the recombination breakpoint [Martin et al. 2011]). Also, the power to detect recombination depends on the effective population size, which in the case of mitochondria depends on the bottleneck levels attributable to mt transmission (Neiman and Taylor 2009). Finally, although in principle there is one homologous site per base available for homologous recombination, there are many more sites available for nonhomologous recombination. This is consistent with the latter type of recombination being more likely to promote gene order changes.

Ectopic recombination is often facilitated by the presence of repeats in both plant and fungal mtDNAs, and it can have serious detrimental effects, including disruption of coding frames or gene expression alteration (Galtier 2011). Different strategies to protect the genome from the negative effects of ectopic recombination have evolved and they are remarkably different in plant and animal mtDNAs (Galtier 2011): although animal mtDNAs avoid the accumulation of repeats and introns at the cost of a higher mutation rate (Lynch et al. 2006), plant mtDNAs have selected for efficient recombination-mediated DNA repair mechanisms, thus explaining the low mutation rate observed in plant mt genomes (Odahara et al. 2009; Davila et al. 2011). Moreover, efficient mismatch repair is often accompanied by gene conversion in plant mtDNA (Davila et al. 2011). In this study, we have not investigated recombination-associated DNA repair mechanisms in fungal mt genomes; it is nevertheless interesting to speculate whether fungi have selected for mtDNA repair mechanisms similar to those found in plants as defense against repeat accumulation. It is known, for instance, that in *P. anserina* the nuclear-encoded gene *grisea* protects mtDNA integrity from the deleterious effects of ectopic recombination (Belcour et al. 1991). It would be particularly interesting to test this hypothesis in other fungal mtDNAs such as those of the sordariomycetes that show evidence of recombination (Kouvelis et al. 2004; Ghikas et al. 2006; Pantou et al. 2008) and high GOC.

The evolution of gene order in fungal mitochondria, particularly in basidiomycetes, suggests a complex interplay of opposing evolutionary forces. Although mt genes tend to be conserved at the sequence level due to their importance in cellular metabolism, here we have shown that in fungal mtDNA gene order is relatively free to vary, and that this variation is probably largely due to recombination (most likely nonhomologous, intramolecular). Indeed, in most studies, the diversity of gene order in mitochondria is taken as evidence of effective recombination. Furthermore, some mtDNA sequence characteristics appear to contribute to gene order variability. In particular, repeats at intergenic sequences tend to increase the probability of recombination, both homologous and nonhomologous, thereby facilitating rearrangement events, in agreement with numerous previous reports (Gordenin et al. 1993; Bi and Liu 1996; Lobachev et al. 1998; Rocha et al. 1999; Waldman et al. 1999; Rocha 2003, 2006; Phadnis et al. 2005; Odahara et al. 2009; Lavrov 2010). Transposable elements and variability of tRNA distribution also appear to contribute to gene order variability although apparently less strongly.

## Supplementary Material

Supplementary figures S1–S3 and tables S1–S5 are available at *Genome Biology and Evolution* online (http://www.gbe.oxfordjournals.org/).

Supplementary Data

## References

[evu028-B1] Adams KL, Palmer JD (2003). Evolution of mitochondrial gene content: gene loss and transfer to the nucleus. Mol Phylogenet Evol..

[evu028-B2] Al-Reedy RM, Malireddy R, Dillman CB, Kennell JC (2012). Comparative analysis of *Fusarium* mitochondrial genomes reveals a highly variable region that encodes an exceptionally large open reading frame. Fungal Genet Biol..

[evu028-B3] Amlacher S (2011). Insight into structure and assembly of the nuclear pore complex by utilizing the genome of a eukaryotic thermophile. Cell.

[evu028-B4] Barr CM, Neiman M, Taylor DR (2005). Inheritance and recombination of mitochondrial genomes in plants, fungi and animals. New Phytol..

[evu028-B5] Bartelli TF, Ferreira RC, Colombo AL, Briones MRS (2013). Intraspecific comparative genomics of *Candida albicans* mitochondria reveals non-coding regions under neutral evolution. Infect Genet Evol..

[evu028-B6] Belcour L, Begel O, Picard M (1991). A site-specific deletion in mitochondrial-DNA of *Podospora* is under the control of nuclear genes. Proc Natl Acad Sci U S A..

[evu028-B7] Bergthorsson U, Richardson AO, Young GJ, Goertzen LR, Palmer JD (2004). Massive horizontal transfer of mitochondrial genes from diverse land plant donors to the basal angiosperm *Amborella*. Proc Natl Acad Sci U S A..

[evu028-B8] Bernt M, Middendorf M (2011). A method for computing an inventory of metazoan mitochondrial gene order rearrangements. BMC Bioinformatics.

[evu028-B9] Bi X, Liu LF (1996). DNA rearrangement mediated by inverted repeats. Proc Natl Acad Sci U S A..

[evu028-B10] Boore JL (1999). Animal mitochondrial genomes. Nucleic Acids Res..

[evu028-B11] Boore JL, Brown WM (1994). Complete DNA-sequence of the mitochondrial genome of the black chiton, *Katharina tunicata*. Genetics.

[evu028-B12] Bouchier C, Ma L, Creno S, Dujon B, Fairhead C (2009). Complete mitochondrial genome sequences of three *Nakaseomyces* species reveal invasion by palindromic GC clusters and considerable size expansion. FEMS Yeast Res..

[evu028-B13] Bruen TC, Philippe H, Bryant D (2006). A simple and robust statistical test for detecting the presence of recombination. Genetics.

[evu028-B14] Bullerwell CE, Burger G, Lang BF (2000). A novel motif for identifying Rps3 homologs in fungal mitochondrial genomes. Trends Biochem Sci..

[evu028-B15] Bullerwell CE, Gray MW (2004). Evolution of the mitochondrial genome: protist connections to animals, fungi and plants. Curr Opin Microbiol..

[evu028-B16] Bullerwell CE, Lang BF (2005). Fungal evolution: the case of the vanishing mitochondrion. Curr Opin Microbiol..

[evu028-B17] Bullerwell CE, Leigh J, Forget L, Lang BF (2003). A comparison of three fission yeast mitochondrial genomes. Nucleic Acids Res..

[evu028-B18] Burger G, Gray MW, Lang BF (2003). Mitochondrial genomes: anything goes. Trends Genet..

[evu028-B19] Capella-Gutierrez S, Silla-Martinez JM, Gabaldon T (2009). trimAl: a tool for automated alignment trimming in large-scale phylogenetic analyses. Bioinformatics.

[evu028-B20] Cardoso MAG, Tambor JHM, Nobrega FG (2007). The mitochondrial genome from the thermal dimorphic fungus *Paracoccidioides brasiliensis*. Yeast.

[evu028-B21] Cedergren R, Lang BF (1985). Probing fungal mitochondrial evolution with transfer-RNA. Biosystems.

[evu028-B22] Darling AE, Mau B, Perna NT (2010). progressiveMauve: multiple genome alignment with gene gain, loss and rearrangement. PLoS One.

[evu028-B23] Davila JI (2011). Double-strand break repair processes drive evolution of the mitochondrial genome in *Arabidopsis*. BMC Biol..

[evu028-B24] Devine SE, Boeke JD (1996). Integration of the yeast retrotransposon Ty1 is targeted to regions upstream of genes transcribed by RNA polymerase III. Genes Dev..

[evu028-B25] Di Rienzi SC, Collingwood D, Raghuraman MK, Brewer BJ (2009). Fragile genomic sites are associated with origins of replication. Genome Biol Evol..

[evu028-B26] Ebersberger I (2012). A consistent phylogenetic backbone for the fungi. Mol Biol Evol..

[evu028-B27] Eldarov MA, Mardanov AV, Beletsky AV, Ravin NV, Skryabin KG (2011). Complete sequence and analysis of the mitochondrial genome of the methylotrophic yeast *Hansenula polymorpha* DL-1. FEMS Yeast Res..

[evu028-B28] Ferandon C (2010). The *Agaricus bisporus cox*1 gene: the longest mitochondrial gene and the largest reservoir of mitochondrial group I Introns. PLoS One.

[evu028-B29] Fischer G, Rocha EPC, Brunet F, Vergassola M, Dujon B (2006). Highly variable rates of genome rearrangements between hemiascomycetous yeast lineages. PLoS Genet..

[evu028-B30] Forget L, Ustinova J, Wang Z, Huss VAR, Lang BF (2002). *Hyaloraphidium curvatum:* a linear mitochondrial genome, tRNA editing, and an evolutionary link to lower fungi. Mol Biol Evol..

[evu028-B31] Formighieri EF (2008). The mitochondrial genome of the phytopathogenic basidiomycete *Moniliophthora perniciosa* is 109 kb in size and contains a stable integrated plasmid. Mycol Res..

[evu028-B32] Gabaldon T, Huynen MA (2003). Reconstruction of the proto-mitochondrial metabolism. Science.

[evu028-B33] Gabaldon T, Huynen MA (2004). Shaping the mitochondrial proteome. Biochim Biophys Acta..

[evu028-B34] Gabaldon T, Huynen MA (2007). From endosymbiont to host-controlled organelle: the hijacking of mitochondrial protein synthesis and metabolism. PLoS Comput Biol..

[evu028-B35] Gabaldon T, Rainey D, Huynen MA (2005). Tracing the evolution of a large protein complex in the eukaryotes, NADH: ubiquinone oxidoreductase (complex I). J Mol Biol..

[evu028-B36] Galtier N (2011). The intriguing evolutionary dynamics of plant mitochondrial DNA. BMC Biol..

[evu028-B37] Ghikas DV, Kouvelis VN, Typas MA (2006). The complete mitochondrial genome of the entomopathogenic fungus *Metarhizium anisopliae* var. *anisopliae*: gene order and trn gene clusters reveal a common evolutionary course for all sordariomycetes, while intergenic regions show variation. Arch Microbiol..

[evu028-B38] Ghikas DV, Kouvelis VN, Typas MA (2010). Phylogenetic and biogeographic implications inferred by mitochondrial intergenic region analyses and ITS1-5.8S-ITS2 of the entomopathogenic fungi *Beauveria bassiana* and *B. brongniartii*. BMC Microbiol..

[evu028-B39] Gordenin DA (1993). Inverted repeats—a source of eukaryotic genomic instability. Mol Cell Biol..

[evu028-B40] Gray MW (1998). Genome structure and gene content in protist mitochondrial DNAs. Nucleic Acids Res..

[evu028-B41] Guindon S, Dufayard JF, Hordijk W, Lefort V, Gascuel O (2009). PhyML: fast and accurate phylogeny reconstruction by maximum likelihood. Infect Genet Evol..

[evu028-B42] Hane JK (2007). Dothideomycete-plant interactions illuminated by genome sequencing and EST analysis of the wheat pathogen *Stagonospora nodorum*. Plant Cell.

[evu028-B43] Haridas S, Gantt JS (2010). The mitochondrial genome of the wood-degrading basidiomycete *Trametes cingulata*. FEMS Microbiol Lett..

[evu028-B44] Hecht J, Grewe F, Knoop V (2011). Extreme RNA editing in coding islands and abundant microsatellites in repeat sequences of *Selaginella moellendorffii* mitochondria: the root of frequent plant mtDNA recombination in early Tracheophytes. Genome Biol Evol..

[evu028-B45] Hilker R, Sickinger C, Pedersen CNS, Stoye J (2012). UniMoG—a unifying framework for genomic distance calculation and sorting based on DCJ. Bioinformatics.

[evu028-B46] Huerta-Cepas J, Dopazo J, Gabaldon T (2010). ETE: a python environment for tree exploration. BMC Bioinformatics.

[evu028-B47] Hughes AL, Friedman R (2004). Transposable element distribution in the yeast genome reflects a role in repeated genomic rearrangement events on an evolutionary time scale. Genetica.

[evu028-B48] Jung PP (2010). Complete mitochondrial genome sequence of the yeast *Pichia farinosa* and comparative analysis of closely related species. Curr Genet..

[evu028-B49] Kerscher S, Durstewitz G, Casaregola S, Gaillardin C, Brandt U (2001). The complete mitochondrial genome of *Yarrowia lipolytica*. Comp Funct Genomics..

[evu028-B50] Kitazaki K, Kubo T (2010). Cost of having the largest mitochondrial genome: evolutionary mechanism of plant mitochondrial genome. J Bot..

[evu028-B51] Kolpakov R, Bana G, Kucherov G (2003). mreps: efficient and flexible detection of tandem repeats in DNA. Nucleic Acids Res..

[evu028-B52] Koszul R (2003). The complete mitochondrial genome sequence of the pathogenic yeast *Candida* (*Torulopsis*) *glabrata*. FEBS Lett..

[evu028-B53] Kouvelis VN, Ghikas DV, Typas MA (2004). The analysis of the complete mitochondrial genome of *Lecanicillium muscarium* (synonym *Verticillium lecanii*) suggests a minimum common gene organization in mtDNAs of sordariomycetes: phylogenetic implications. Fungal Genet Biol..

[evu028-B54] Kueberl A (2011). High-quality genome sequence of *Pichia pastoris* CBS7435. J Biotechnol..

[evu028-B55] Kurabayashi A, Ueshima R (2000). Complete sequence of the mitochondrial DNA of the primitive opisthobranch gastropod *Pupa strigosa:* systematic implication of the genome organization. Mol Biol Evol..

[evu028-B56] Lambowitz AM, Perlman PS (1990). Involvement of aminoacyl-transfer RNA-synthetases and other proteins in group-I and group-II intron-splicing. Trends Biochem Sci..

[evu028-B57] Lang BF, Laforest M-J, Burger G (2007). Mitochondrial introns: a critical view. Trends Genet..

[evu028-B58] Lavrov DV (2010). Rapid proliferation of repetitive palindromic elements in mtDNA of the endemic Baikalian sponge *Lubomirskia baicalensis*. Mol Biol Evol..

[evu028-B59] Lavrov DV, Boore JL, Brown WM (2002). Complete mtDNA sequences of millipedes suggest a new model for mitochondrial gene rearrangements: duplication and nonrandom loss. Mol Biol Evol..

[evu028-B60] Lazowska J, Jacq C, Slonimski PP (1980). Sequence of introns and flanking exons in wild-type and box3 mutants of cytochrome-B reveals an interlaced splicing protein coded by an intron. Cell.

[evu028-B61] Levings CS, Brown GG (1989). Molecular-biology of plant-mitochondria. Cell.

[evu028-B62] Litter J, Keszthelyi A, Hamari Z, Pfeiffer I, Kucsera J (2005). Differences in mitochondrial genome organization of *Cryptococcus neoformans* strains. Antonie Van Leeuwenhoek..

[evu028-B63] Liu Y, Xue J-Y, Wang B, Li L, Qiu Y-L (2011). The mitochondrial genomes of the early land plants *Treubia lacunosa* and *Anomodon rugelii:* dynamic and conservative evolution. PLoS One.

[evu028-B64] Lobachev KS (1998). Factors affecting inverted repeat stimulation of recombination and deletion in *Saccharomyces cerevisiae*. Genetics.

[evu028-B65] Lucking R, Huhndorf S, Pfister DH, Plata ER, Lumbsch HT (2009). Fungi evolved right on track. Mycologia.

[evu028-B66] Lynch M, Blanchard JL (1998). Deleterious mutation accumulation in organelle genomes. Genetica.

[evu028-B67] Lynch M, Koskella B, Schaack S (2006). Mutation pressure and the evolution of organelle genomic architecture. Science.

[evu028-B68] Marcet-Houben M, Gabaldon T (2009). The tree versus the forest: the fungal tree of life and the topological diversity within the yeast phylome. PLoS One.

[evu028-B69] Martin DP, Lemey P, Posada D (2011). Analysing recombination in nucleotide sequences. Mol Ecol Resour..

[evu028-B70] Martin DP (2010). RDP3: a flexible and fast computer program for analyzing recombination. Bioinformatics.

[evu028-B71] Maydt J, Lengauer T (2006). Recco: recombination analysis using cost optimization. Bioinformatics.

[evu028-B72] Nabholz B, Glemin S, Galtier N (2009). The erratic mitochondrial clock: variations of mutation rate, not population size, affect mtDNA diversity across birds and mammals. BMC Evol Biol..

[evu028-B73] Neiman M, Taylor DR (2009). The causes of mutation accumulation in mitochondrial genomes. Proc R Soc B Biol Sci..

[evu028-B74] Notredame C, Higgins DG, Heringa J (2000). T-Coffee: a novel method for fast and accurate multiple sequence alignment. J Mol Biol..

[evu028-B75] Odahara M, Kuroiwa H, Kuroiwa T, Sekine Y (2009). Suppression of repeat-mediated gross mitochondrial genome rearrangements by RecA in the moss *Physcomitrella patens*. Plant Cell.

[evu028-B76] Palmer JD (2000). Dynamic evolution of plant mitochondrial genomes: mobile genes and introns and highly variable mutation rates. Proc Natl Acad Sci U S A..

[evu028-B77] Pantou MP, Kouvelis VN, Typas MA (2008). The complete mitochondrial genome of *Fusarium oxysporum*: insights into fungal mitochondrial evolution. Gene.

[evu028-B78] Paquin B, Lang BF (1996). The mitochondrial DNA of *Allomyces macrogynus:* the complete genomic sequence from an ancestral fungus. J Mol Biol..

[evu028-B79] Paquin B (1997). The fungal mitochondrial genome project: evolution of fungal mitochondrial genomes and their gene expression. Curr Genet..

[evu028-B80] Pellenz S, Harington A, Dujon B, Wolf K, Schafer B (2002). Characterization of the I-SpomI endonuclease from fission yeast: insights into the evolution of a group I intron-encoded homing endonuclease. J Mol Evol..

[evu028-B81] Perseke M (2008). Evolution of mitochondrial gene orders in echinoderms. Mol Phylogenet Evol..

[evu028-B82] Phadnis N, Sia RA, Sia EA (2005). Analysis of repeat-mediated deletions in the mitochondrial genome of *Saccharomyces cerevisiae*. Genetics.

[evu028-B83] Posada D, Crandall KA (2001). Evaluation of methods for detecting recombination from DNA sequences: computer simulations. Proc Natl Acad Sci U S A..

[evu028-B84] Prochazka E, Polakova S, Piskur J, Sulo P (2010). Mitochondrial genome from the facultative anaerobe and petite-positive yeast *Dekkera bruxellensis* contains the NADH dehydrogenase subunit genes. FEMS Yeast Res..

[evu028-B116] http://www.R-project.org/.

[evu028-B85] Rawlings TA, Collins TM, Bieler R (2001). A major mitochondrial gene rearrangement among closely related species. Mol Biol Evol..

[evu028-B86] Rocha EPC (2003). DNA repeats lead to the accelerated loss of gene order in bacteria. Trends Genet..

[evu028-B87] Rocha EPC (2006). Inference and analysis of the relative stability of bacterial chromosomes. Mol Biol Evol..

[evu028-B88] Rocha EPC, Danchin A, Viari A (1999). Functional and evolutionary roles of long repeats in prokaryotes. Res Microbiol..

[evu028-B89] Rokas A, Ladoukakis E, Zouros E (2003). Animal mitochondrial DNA recombination revisited. Trends Ecol Evol..

[evu028-B90] Saccone C (2002). Mitochondrial DNA in metazoa: degree of freedom in a frozen event. Gene.

[evu028-B91] Sacerdot C (2008). Promiscuous DNA in the nuclear genomes of hemiascomycetous yeasts. FEMS Yeast Res..

[evu028-B92] Sanderson MJ (2003). r8s: inferring absolute rates of molecular evolution and divergence times in the absence of a molecular clock. Bioinformatics.

[evu028-B93] Seif ER, Forget L, Martin NC, Lang BF (2003). Mitochondrial RNase P RNAs in ascomycete fungi: lineage-specific variations in RNA secondary structure. RNA.

[evu028-B94] Seif E (2005). Comparative mitochondrial genomics in zygomycetes: bacteria-like RNase P RNAs, mobile elements and a close source of the group I intron invasion in angiosperms. Nucleic Acids Res..

[evu028-B95] Sloan DB (2012). Rapid evolution of enormous, multichromosomal genomes in flowering plant mitochondria with exceptionally high mutation rates. PLoS Biol..

[evu028-B96] Specht CA, Novotny CP, Ullrich RC (1992). Mitochondrial DNA of *Schizophyllum commune—*restriction map, genetic map and mode of inheritance. Curr Genet..

[evu028-B97] Stamatakis A (2006). RAxML-VI-HPC: maximum likelihood-based phylogenetic analyses with thousands of taxa and mixed models. Bioinformatics.

[evu028-B98] Stone CL, Posada Buitrago ML, Boore JL, Frederick RD (2010). Analysis of the complete mitochondrial genome sequences of the soybean rust pathogens *Phakopsora pachyrhizi* and *P*. meibomiae. Mycologia.

[evu028-B99] Tamames J (2001). Evolution of gene order conservation in prokaryotes. Genome Biol..

[evu028-B100] Taylor JW, Berbee ML (2006). Dating divergences in the fungal tree of life: review and new analyses. Mycologia.

[evu028-B101] Tesler G (2002). GRIMM: genome rearrangements web server. Bioinformatics.

[evu028-B102] Torriani SFF, Goodwin SB, Kema GHJ, Pangilinan JL, McDonald BA (2008). Intraspecific comparison and annotation of two complete mitochondrial genome sequences from the plant pathogenic fungus *Mycosphaerella graminicola*. Fungal Genet Biol..

[evu028-B103] Tuller T (2011). Association between translation efficiency and horizontal gene transfer within microbial communities. Nucleic Acids Res..

[evu028-B104] van Diepeningen AD (2010). Mitochondrial recombination increases with age in *Podospora anserina*. Mech Ageing Dev..

[evu028-B105] Vaughn JC, Mason MT, Sperwhitis GL, Kuhlman P, Palmer JD (1995). Fungal origin by horizontal transfer of a plant mitochondrial group-I intron in the chimeric *coxi* gene of *Peperomia*. J Mol Evol..

[evu028-B106] Vlcek C, Marande W, Teijeiro S, Lukes J, Burger G (2011). Systematically fragmented genes in a multipartite mitochondrial genome. Nucleic Acids Res..

[evu028-B107] Waldman AS, Tran H, Goldsmith EC, Resnick MA (1999). Long inverted repeats are an at-risk motif for recombination in mammalian cells. Genetics.

[evu028-B108] Wang Y, Zeng F, Hon CC, Zhang Y, Leung FCC (2008). The mitochondrial genome of the Basidiomycete fungus *Pleurotus ostreatus* (oyster mushroom). FEMS EMS Microbiol Lett..

[evu028-B109] Wolfe KH, Li WH, Sharp PM (1987). Rates of nucleotide substitution vary greatly among plant mitochondrial, chloroplast and nuclear DNAs. Proc Natl Acad Sci U S A..

[evu028-B110] Wolfe KH, Shields DC (1997). Molecular evidence for an ancient duplication of the entire yeast genome. Nature.

[evu028-B111] Woo PCY (2003). The mitochondrial genome of the thermal dimorphic fungus *Penicillium marneffei* is more closely related to those of molds than yeasts. FEBS Lett..

[evu028-B112] Wu Y (2009). Recent dermatophyte divergence revealed by comparative and phylogenetic analysis of mitochondrial genomes. BMC Genomics.

[evu028-B113] Xavier BB, Miao VPW, Jonsson ZO, Andresson OS (2012). Mitochondrial genomes from the lichenized fungi *Peltigera membranacea* and *Peltigera malacea:* features and phylogeny. Fungal Biol..

[evu028-B114] Yamazaki N (1997). Evolution of pulmonate gastropod mitochondrial genomes: comparisons of gene organizations of *Euhadra*, *Cepaea* and *Albinaria* and implications of unusual tRNA secondary structures. Genetics.

[evu028-B115] Zivanovic Y, Wincker P, Vacherie B, Bolotin-Fukuhara M, Fukuhara H (2005). Complete nucleotide sequence of the mitochondrial DNA from *Kluyveromyces lactis*. FEMS Yeast Res..

