# Supplementary material for: High Variability of Mitochondrial Gene Order among Fungi
Source: Genome Biol Evol. 2014 Feb 6;6(2):451–65. doi: 10.1093/gbe/evu028 (PMC3942027; doi:10.1093/gbe/evu028)
Supplement: Supplementary Data [file supp_6_2_451__index.html]

High variability of mitochondrial gene order among fungi — High Variability of Mitochondrial Gene Order among Fungi — Supplementary Data 

# High Variability of Mitochondrial Gene Order among Fungi

## Supplementary Data

files

**Files in this Data Supplement:**

- Supplementary Data - pdf file
- Supplementary Data - pdf file
- Supplementary Data - pdf file
