# Supplementary figures and images for: High Variability of Mitochondrial Gene Order among Fungi
Source: Genome Biol Evol. 2014 Feb 6;6(2):451–65. doi: 10.1093/gbe/evu028 (PMC3942027; doi:10.1093/gbe/evu028)

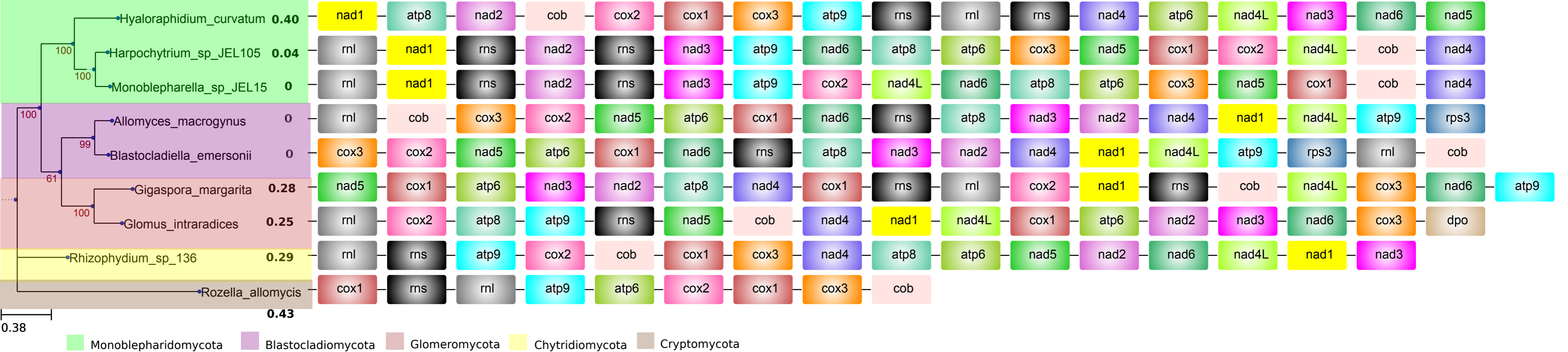

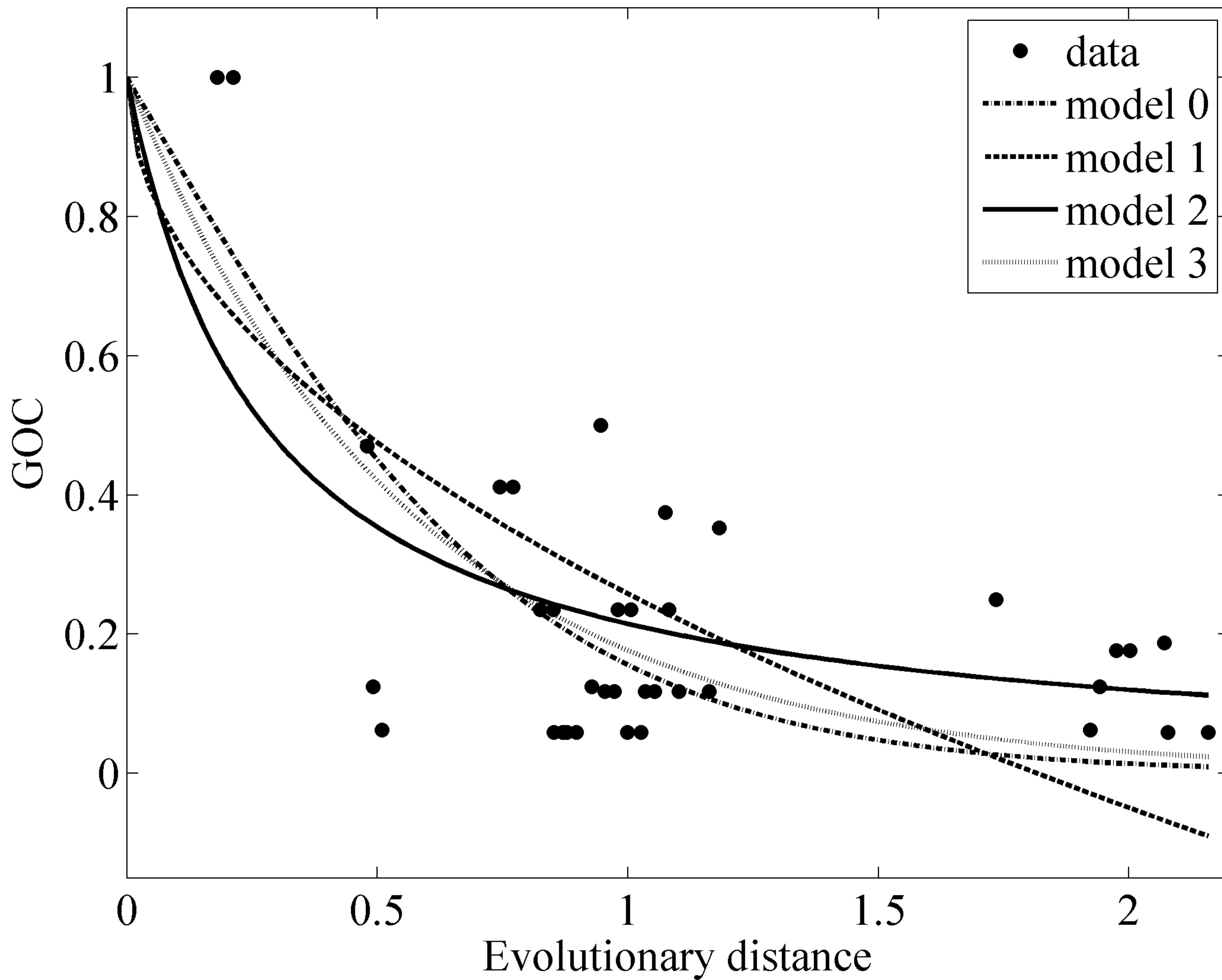

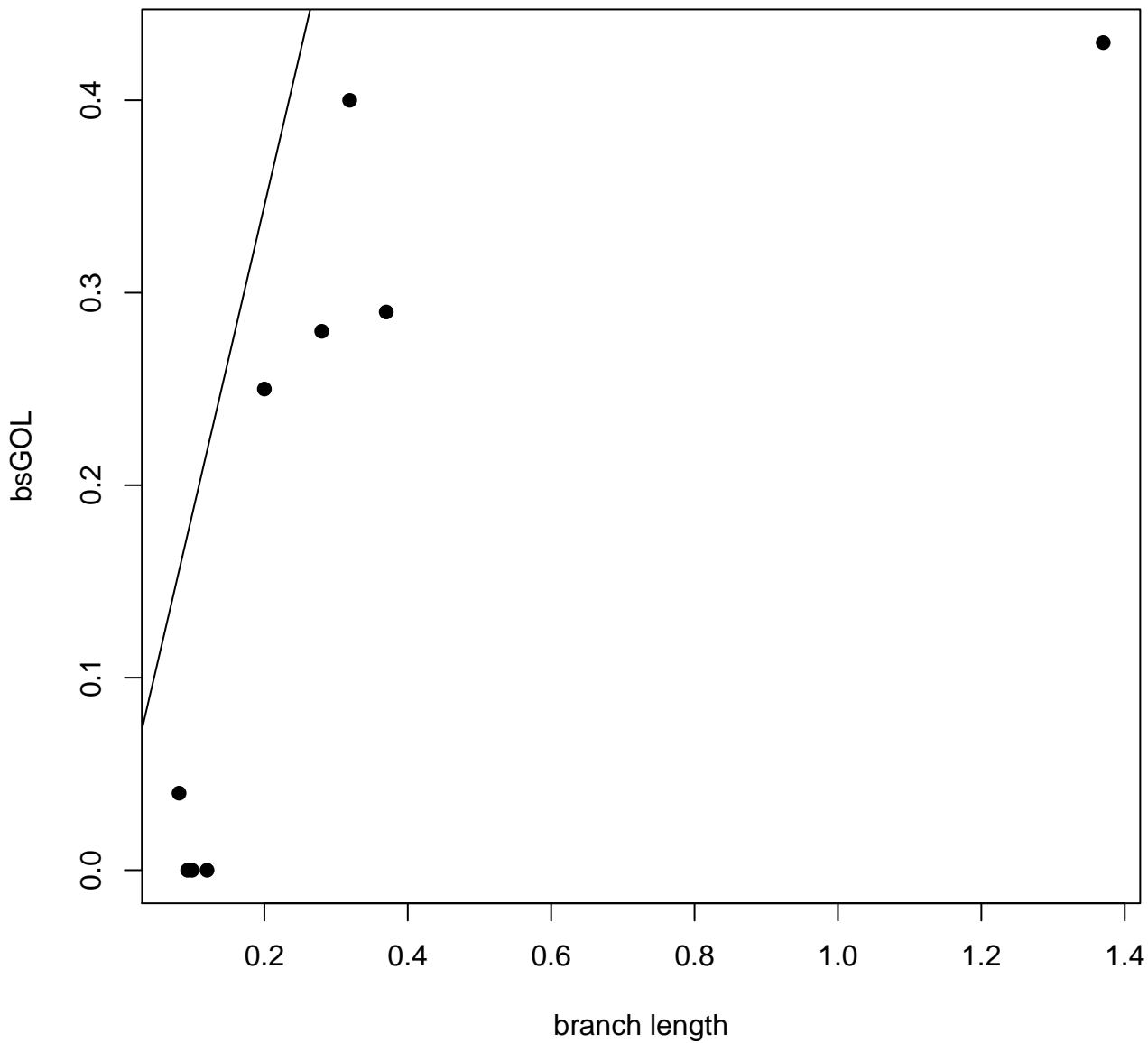

Supplement: Supplementary Data [file supp_evu028_SupplementaryFigures.pdf]
