# Supplementary material for: High Variability of Mitochondrial Gene Order among Fungi
Source: Genome Biol Evol. 2014 Feb 6;6(2):451–65. doi: 10.1093/gbe/evu028 (PMC3942027; doi:10.1093/gbe/evu028)
Supplement: Supplementary Data [file supp_evu028_SupplementaryTables.pdf]

Supplementary Table 1

Pairwise gene order conservation and loss between all pairs of dikarya fungi

Species	GOC[a]	GOL[b]	p-val	distance[c]
Allomyces-Arthroderma	0.117647059	0.882352941	0.1666	1.4425503
Allomyces-Beauveria	0.352941176	0.647058824	0.7136	1.59665979
Allomyces-Calbicans	0.117647059	0.882352941	0.1171	1.996212
Allomyces-Cglabrata	0.176470588	0.823529412	0.7838	1.6234143
Allomyces-Chaetomium	0.352941176	0.647058824	0.8569	1.4895972
Allomyces-Cordyceps	0.352941176	0.647058824	0.6831	1.60270418
Allomyces-Crypto	0.352941176	0.647058824	0.7097	1.7222499
Allomyces-Debaryomyces	0.235294118	0.764705882	0.4015	2.100167
Allomyces-Dekkera	0.444444444	0.555555556	0.9019	1.908351
Allomyces-Fusarium	0.352941176	0.647058824	0.6782	1.5270712
Allomyces-Gibberella	0.235294118	0.764705882	0.4431	1.529134
Allomyces-Kluyveromyces	0.117647059	0.882352941	0.3319	1.580022
Allomyces-Lecanicillium	0.352941176	0.647058824	0.6791	1.640755
Allomyces-Metarhizium	0.352941176	0.647058824	0.6791	1.6086911
Allomyces-Micosphaerella	0.117647059	0.882352941	0.1536	1.4208626
Allomyces-Microsporum	0.117647059	0.882352941	0.1735	1.4418994
Allomyces-Millerozyma	0.235294118	0.764705882	0.4098	2.115312
Allomyces-Monilio	0.210526316	0.789473684	0.4402	1.4413184
Allomyces-mviol_mt	0.1	0.9	0.1496	1.320071
Allomyces-Nakaseomyces	0.117647059	0.882352941	0.3296	1.7325985
Allomyces-Ogataea	0.470588235	0.529411765	0.8789	2.024812
Allomyces-P_paky	0.529411765	0.470588235	0.9564	1.771911
Allomyces-Paracoccidioides	0.117647059	0.882352941	0.1339	1.5445167
Allomyces-Peltigera	0.315789474	0.684210526	0.7408	1.4946906
Allomyces-Penicillium	0.235294118	0.764705882	0.4105	1.4263482
Allomyces-Phaeosphaeria	0.352941176	0.647058824	0.9176	1.4726326
Allomyces-Pichia	0.352941176	0.647058824	0.79	1.751849
Allomyces-Pleurotus	0.210526316	0.789473684	0.4368	1.3755134
Allomyces-Podospora	0.333333333	0.666666667	0.7263	1.4816725
Allomyces-Rhizophydium	0.235294118	0.764705882	0.4105	1.812442
Allomyces-Schizosaccharomyces	0.235294118	0.764705882	0.7142	1.759868
Allomyces-Scommu	0.117647059	0.882352941	0.1024	1.498071
Allomyces-T_indi	0.117647059	0.882352941	0.1182	1.3031909
Allomyces-Tramet	0.176470588	0.823529412	0.3593	1.267139
Allomyces-Umaydis	0.352941176	0.647058824	0.6758	1.2432229
Allomyces-Vanderwaltozyma	0.117647059	0.882352941	0.3796	1.6580833
Allomyces-Yarrowia	0.117647059	0.882352941	0.1537	1.70706
Arthroderma-Beauveria	0.5	0.5	0.9477	0.64619809
Arthroderma-Calbicans	0.25	0.75	0.4803	1.6555863
Arthroderma-Cglabrata	0.133333333	0.866666667	0.3198	1.2827886
Arthroderma-Chaetomium	0.4	0.6	0.884	0.5391355
Arthroderma-Cordyceps	0.470588235	0.529411765	0.9553	0.65224248
Arthroderma-Crypto	0.125	0.875	0.2029	1.5923462

Arthroderma-Debaryomyces	0.375	0.625	0.794	1.7595413
Arthroderma-Dekkera	0.222222222	0.777777778	0.5471	1.5677253
Arthroderma-Fusarium	0.470588235	0.529411765	0.9614	0.5766095
Arthroderma-Gibberella	0.533333333	0.466666667	0.9289	0.5786723
Arthroderma-Kluyveromyces	0.066666667	0.933333333	0	1.2393963
Arthroderma-Lecanicillium	0.470588235	0.529411765	0.9561	0.6902933
Arthroderma-Metarhizium	0.470588235	0.529411765	0.9596	0.6582294
Arthroderma-Micosphaerella	0.4	0.6	0.7232	0.4704009
Arthroderma-Microsporum	1	0	0.9999	0.0384623
Arthroderma-Millerozyma	0.125	0.875	0.1481	1.7746863
Arthroderma-Monilio	0.315789474	0.684210526	0.8628	1.3114147
Arthoderma-mviol_mt	0.1	0.9	0.2278	1.1901673
Arthroderma-Nakaseomyces	0.066666667	0.933333333	0	1.3919728
Arthroderma-Ogataea	0.470588235	0.529411765	0.959	1.6841863
Arthroderma-P_paky	0.117647059	0.882352941	0.1731	1.6420073
Arthroderma-Paracoccidioides	0.866666667	0.133333333	0.9997	0.3772536
Arthroderma-Peltigera	0.210526316	0.789473684	0.5922	0.5442289
Arthroderma-Penicillium	0.5	0.5	0.9479	0.3382881
Arthroderma-Phaeosphaeria	0.133333333	0.866666667	0.2108	0.5221709
Arthroderma-Pichia	0.466666667	0.533333333	0.8796	1.4112233
Arthroderma-Pleurotus	0.105263158	0.894736842	0.2126	1.2456097
Arthroderma-Podospora	0.277777778	0.722222222	0.8685	0.5312108
Arthroderma-Rhizophydium	0.375	0.625	0.7879	2.0635483
Arthroderma-Schizosaccharomyces	0.133333333	0.866666667	0.4319	1.4192423
Arthroderma-Scommu	0.235294118	0.764705882	0.5168	1.3681673
Arthroderma-T_indi	0.125	0.875	0.1517	1.1732872
Arthroderma-Tramet	0.235294118	0.764705882	0.531	1.1372353
Arthroderma-Umaydis	0.235294118	0.764705882	0.5281	1.1133192
Arthroderma-Vanderwaltozyma	0.066666667	0.933333333	0	1.3174576
Arthroderma-Yarrowia	0.4	0.6	0.7181	1.3664343
Beauveria-Calbicans	0.375	0.625	0.7537	1.80969579
Beauveria-Cglabrata	0.375	0.625	0.9842	1.43689809
Beauveria-Chaetomium	0.6875	0.3125	0.9993	0.45219559
Beauveria-Cordyceps	0.941176471	0.058823529	1	0.01161087
Beauveria-Crypto	0.25	0.75	0.4428	1.74645569
Beauveria-Debaryomyces	0.375	0.625	0.7496	1.91365079
Beauveria-Dekkera	0.222222222	0.777777778	0.4431	1.72183479
Beauveria-Fusarium	0.941176471	0.058823529	1	0.21023159
Beauveria-Gibberella	0.9375	0.0625	1	0.21229439
Beauveria-Kluyveromyces	0.125	0.875	0.4012	1.39350579
Beauveria-Lecanicillium	0.941176471	0.058823529	1	0.06160259
Beauveria-Metarhizium	0.941176471	0.058823529	1	0.23685869
Beauveria-Micosphaerella	0.625	0.375	0.99	0.55798059
Beauveria-Microsporum	0.5	0.5	0.9462	0.64554719
Beauveria-Millerozyma	0.375	0.625	0.7561	1.92879579

Beauveria-Monilio	0.315789474	0.684210526	0.7769	1.46552419
Beauveria-mviol_mt	0.2	0.8	0.5028	1.34427679
Beauveria-Nakaseomyces	0.25	0.75	0.8478	1.54608229
Beauveria-Ogataea	0.411764706	0.588235294	0.8758	1.83829579
Beauveria-P_paky	0.352941176	0.647058824	0.7153	1.79611679
Beauveria-Paracoccidioides	0.625	0.375	0.993	0.74816449
Beauveria-Peltigera	0.684210526	0.315789474	0.9996	0.63180859
Beauveria-Penicillium	0.5625	0.4375	0.9766	0.62999599
Beauveria-Phaeosphaeria	0.25	0.75	0.6293	0.60975059
Beauveria-Pichia	0.625	0.375	0.9881	1.56533279
Beauveria-Pleurotus	0.210526316	0.789473684	0.4763	1.39971919
Beauveria-Podospora	0.666666667	0.333333333	0.9992	0.44427089
Beauveria-Rhizophydium	0.4375	0.5625	0.9007	2.21765779
Beauveria-Schizosaccharomyces	0.25	0.75	0.7912	1.57335179
Beauveria-Scommu	0.352941176	0.647058824	0.7134	1.52227679
Beauveria-T_indi	0.625	0.375	0.9864	1.32739669
Beauveria-Tramet	0.411764706	0.588235294	0.8732	1.29134479
Beauveria-Umaydis	0.470588235	0.529411765	0.9032	1.26742869
Beauveria-Vanderwaltozyma	0.125	0.875	0.3539	1.47156709
Beauveria-Yarrowia	0.75	0.25	0.9987	1.52054379
Calbicans-Cglabrata	0.25	0.75	0.7965	0.9861223
Calbicans-Chaetomium	0.375	0.625	0.8985	1.7026332
Calbicans-Cordyceps	0.352941176	0.647058824	0.7149	1.81574018
Calbicans-Crypto	0.5	0.5	0.9311	2.1460079
Calbicans-Debaryomyces	0.6875	0.3125	0.9907	0.585695
Calbicans-Dekkera	0.611111111	0.388888889	0.9949	1.271059
Calbicans-Fusarium	0.352941176	0.647058824	0.7105	1.7401072
Calbicans-Gibberella	0.375	0.625	0.7925	1.74217
Calbicans-Kluyveromyces	0.4375	0.5625	0.9915	0.94273
Calbicans-Lecanicillium	0.352941176	0.647058824	0.7188	1.853791
Calbicans-Metarhizium	0.352941176	0.647058824	0.7198	1.8217271
Calbicans-Micosphaerella	0.5	0.5	0.929	1.6338986
Calbicans-Microsporum	0.25	0.75	0.4892	1.6549354
Calbicans-Millerozyma	0.75	0.25	0.9966	0.60084
Calbicans-Monilio	0.315789474	0.684210526	0.7759	1.8650764
Calbicans-mviol_mt	0.45	0.55	0.9842	1.743829
Calbicans-Nakaseomyces	0.3125	0.6875	0.9354	1.0953065
Calbicans-Ogataea	0.352941176	0.647058824	0.7096	1.38752
Calbicans-P_paky	0.470588235	0.529411765	0.903	2.195669
Calbicans-Paracoccidioides	0.375	0.625	0.7135	1.7575527
Calbicans-Peltigera	0.368421053	0.631578947	0.9125	1.7077266
Calbicans-Penicillium	0.25	0.75	0.3735	1.6393842
Calbicans-Phaeosphaeria	0.25	0.75	0.6282	1.6856686
Calbicans-Pichia	0.5625	0.4375	0.9762	1.114557
Calbicans-Pleurotus	0.210526316	0.789473684	0.4685	1.7992714
Calbicans-Podospora	0.222222222	0.777777778	0.453	1.6947085

Calbicans-Rhizophydium	0.125	0.875	0.1008	2.61721
Calbicans-Schizosaccharomyces	0.125	0.875	0.2641	1.972904
Calbicans-Scommu	0.352941176	0.647058824	0.7097	1.921829
Calbicans-T_indi	0.8125	0.1875	0.999	1.7269489
Calbicans-Tramet	0.235294118	0.764705882	0.4095	1.690897
Calbicans-Umaydis	0.529411765	0.470588235	0.965	1.6669809
Calbicans-Vanderwaltozyma	0.3125	0.6875	0.956	1.0207913
Calbicans-Yarrowia	0.5625	0.4375	0.9765	1.414898
Cglabrata-Chaetomium	0.166666667	0.833333333	0.6646	1.3298355
Cglabrata-Cordyceps	0.352941176	0.647058824	0.9836	1.44294248
Cglabrata-Crypto	0.25	0.75	0.8055	1.7732102
Cglabrata-Debaryomyces	0.25	0.75	0.7937	1.0900773
Cglabrata-Dekkera	0.055555556	0.944444444	0	0.8982613
Cglabrata-Fusarium	0.352941176	0.647058824	0.9862	1.3673095
Cglabrata-Gibberella	0.4	0.6	0.9769	1.3693723
Cglabrata-Kluyveromyces	0.5	0.5	0.8333	0.3178843
Cglabrata-Lecanicillium	0.352941176	0.647058824	0.9809	1.4809933
Cglabrata-Metarhizium	0.352941176	0.647058824	0.9859	1.4489294
Cglabrata-Micosphaerella	0.285714286	0.714285714	0.7423	1.2611009
Cglabrata-Microsporum	0.133333333	0.866666667	0.3239	1.2821377
Cglabrata-Millerozyma	0.25	0.75	0.8069	1.1052223
Cglabrata-Monilio	0.105263158	0.894736842	0.4245	1.4922787
Cglabrata-mviol_mt	0.2	0.8	0.8667	1.3710313
Cglabrata-Nakaseomyces	0.75	0.25	0.7807	0.2905538
Cglabrata-Ogataea	0.117647059	0.882352941	0.371	1.0147223
Cglabrata-P_paky	0.235294118	0.764705882	0.8193	1.8228713
Cglabrata-Paracoccidioides	0.266666667	0.733333333	0.7743	1.384755
Cglabrata-Peltigera	0.210526316	0.789473684	0.8583	1.3349289
Cglabrata-Penicillium	0.125	0.875	0.3481	1.2665865
Cglabrata-Phaeosphaeria	0.2	0.8	0.4194	1.3128709
Cglabrata-Pichia	0.357142857	0.642857143	0.9356	0.7417593
Cglabrata-Pleurotus	0.105263158	0.894736842	0.4227	1.4264737
Cglabrata-Podospora	0.111111111	0.888888889	0.5182	1.3219108
Cglabrata-Rhizophydium	0.375	0.625	0.9814	2.2444123
Cglabrata-Schizosaccharomyces	0.222222222	0.777777778	0.1257	1.6001063
Cglabrata-Scommu	0.235294118	0.764705882	0.8211	1.5490313
Cglabrata-T_indi	0.25	0.75	0.8023	1.3541512
Cglabrata-Tramet	0.117647059	0.882352941	0.3658	1.3180993
Cglabrata-Umaydis	0.294117647	0.705882353	0.9645	1.2941832
Cglabrata-Vanderwaltozyma	0.857142857	0.142857143	0.667	0.312835
Cglabrata-Yarrowia	0.357142857	0.642857143	0.9386	1.0421003
Chaetomium-Cordyceps	0.647058824	0.352941176	0.9998	0.45823998
Chaetomium-Crypto	0.125	0.875	0.2362	1.6393931
Chaetomium-Debaryomyces	0.375	0.625	0.9007	1.8065882
Chaetomium-Dekkera	0.277777778	0.722222222	0.8429	1.6147722

Chaetomium-Fusarium	0.647058824	0.352941176	0.9999	0.382607
Chaetomium-Gibberella	0.8	0.2	1	0.3846698
Chaetomium-Kluyveromyces	0.166666667	0.833333333	0.7624	1.2864432
Chaetomium-Lecanicillium	0.647058824	0.352941176	0.9998	0.4962908
Chaetomium-Metarhizium	0.647058824	0.352941176	0.9996	0.4642269
Chaetomium-Micosphaerella	0.571428571	0.428571429	0.9756	0.450918
Chaetomium-Microsporum	0.4	0.6	0.8809	0.5384846
Chaetomium-Millerozyma	0.4375	0.5625	0.9719	1.8217332
Chaetomium-Monilio	0.315789474	0.684210526	0.8908	1.3584616
Chaetomium-mviol_mt	0.3	0.7	0.9075	1.2372142
Chaetomium-Nakaseomyces	0.166666667	0.833333333	0.7181	1.4390197
Chaetomium-Ogataea	0.352941176	0.647058824	0.8562	1.7312332
Chaetomium-P_paky	0.352941176	0.647058824	0.8589	1.6890542
Chaetomium-Paracoccidioides	0.466666667	0.533333333	0.9637	0.6411019
Chaetomium-Peltigera	0.473684211	0.526315789	0.998	0.524746
Chaetomium-Penicillium	0.375	0.625	0.9029	0.5229334
Chaetomium-Phaeosphaeria	0.333333333	0.666666667	0.823	0.502688
Chaetomium-Pichia	0.571428571	0.428571429	0.9777	1.4582702
Chaetomium-Pleurotus	0.210526316	0.789473684	0.6192	1.2926566
Chaetomium-Podospora	0.444444444	0.555555556	0.9927	0.1962353
Chaetomium-Rhizophydium	0.1875	0.8125	0.6002	2.1105952
Chaetomium-Schizosaccharomyces	0.083333333	0.916666667	0	1.4662892
Chaetomium-Scommu	0.235294118	0.764705882	0.5657	1.4152142
Chaetomium-T_indi	0.375	0.625	0.894	1.2203341
Chaetomium-Tramet	0.470588235	0.529411765	0.9746	1.1842822
Chaetomium-Umaydis	0.352941176	0.647058824	0.8621	1.1603661
Chaetomium-Vanderwaltozyma	0.166666667	0.833333333	0.6678	1.3645045
Chaetomium-Yarrowia	0.571428571	0.428571429	0.9762	1.4134812
Cordyceps-Crypto	0.352941176	0.647058824	0.7151	1.75250008
Cordyceps-Debaryomyces	0.352941176	0.647058824	0.7141	1.91969518
Cordyceps-Dekkera	0.222222222	0.777777778	0.4	1.72787918
Cordyceps-Fusarium	1	0	1	0.21627598
Cordyceps-Gibberella	0.882352941	0.117647059	1	0.21833878
Cordyceps-Kluyveromyces	0.117647059	0.882352941	0.3347	1.39955018
Cordyceps-Lecanicillium	1	0	1	0.06764698
Cordyceps-Metarhizium	1	0	1	0.24290308
Cordyceps-Micosphaerella	0.588235294	0.411764706	0.9927	0.56402498
Cordyceps-Microsporum	0.470588235	0.529411765	0.9528	0.65159158
Cordyceps-Millerozyma	0.352941176	0.647058824	0.7133	1.93484018
Cordyceps-Monilio	0.315789474	0.684210526	0.7424	1.47156858
Cordyceps-mviol_mt	0.2	0.8	0.4594	1.35032118
Cordyceps-Nakaseomyces	0.294117647	0.705882353	0.9399	1.55212668
Cordyceps-Ogataea	0.411764706	0.588235294	0.8528	1.84434018
Cordyceps-P_paky	0.352941176	0.647058824	0.6733	1.80216118
Cordyceps-Paracoccidioides	0.647058824	0.352941176	0.9973	0.75420888

Cordyceps-Peltigera	0.736842105	0.263157895	1	0.63785298
Cordyceps-Penicillium	0.588235294	0.411764706	0.981	0.63604038
Cordyceps-Phaeosphaeria	0.235294118	0.764705882	0.6607	0.61579498
Cordyceps-Pichia	0.529411765	0.470588235	0.9843	1.57137718
Cordyceps-Pleurotus	0.210526316	0.789473684	0.4362	1.40576358
Cordyceps-Podospora	0.722222222	0.277777778	0.9992	0.45031528
Cordyceps-Rhizophydium	0.411764706	0.588235294	0.8797	2.22370218
Cordyceps-Schizosaccharomyces	0.117647059	0.882352941	0.2882	1.57939618
Cordyceps-Scommu	0.470588235	0.529411765	0.8878	1.52832118
Cordyceps-T_indi	0.705882353	0.294117647	0.9972	1.33344108
Cordyceps-Tramet	0.411764706	0.588235294	0.8454	1.29738918
Cordyceps-Umaydis	0.470588235	0.529411765	0.888	1.27347308
Cordyceps-Vanderwaltozyma	0.117647059	0.882352941	0.373	1.47761148
Cordyceps-Yarrowia	0.647058824	0.352941176	0.9985	1.52658818
Crypto-Debaryomyces	0.25	0.75	0.4459	2.2499629
Crypto-Dekkera	0.222222222	0.777777778	0.4381	2.0581469
Crypto-Fusarium	0.352941176	0.647058824	0.71	1.6768671
Crypto-Gibberella	0.125	0.875	0.1525	1.6789299
Crypto-Kluyveromyces	0.125	0.875	0.3097	1.7298179
Crypto-Lecanicillium	0.352941176	0.647058824	0.7129	1.7905509
Crypto-Metarhizium	0.352941176	0.647058824	0.7042	1.758487
Crypto-Micosphaerella	0.125	0.875	0.1798	1.5706585
Crypto-Microsporum	0.125	0.875	0.1968	1.5916953
Crypto-Millerozyma	0.125	0.875	0.134	2.2651079
Crypto-Monilio	0.315789474	0.684210526	0.7744	1.3081143
Crypto-mviol_mt	0.4	0.6	0.9474	1.0497915
Crypto-Nakaseomyces	0.25	0.75	0.7395	1.8823944
Crypto-Ogataea	0.352941176	0.647058824	0.7202	2.1746079
Crypto-P_paky	0.352941176	0.647058824	0.7193	1.5016315
Crypto-Paracoccidioides	0.125	0.875	0.162	1.6943126
Crypto-Peltigera	0.526315789	0.473684211	0.9883	1.6444865
Crypto-Penicillium	0.5625	0.4375	0.9789	1.5761441
Crypto-Phaeosphaeria	0.25	0.75	0.7458	1.6224285
Crypto-Pichia	0.25	0.75	0.5304	1.9016449
Crypto-Pleurotus	0.210526316	0.789473684	0.4707	1.2423093
Crypto-Podospora	0.222222222	0.777777778	0.455	1.6314684
Crypto-Rhizophydium	0.125	0.875	0.1353	2.3432479
Crypto-Schizosaccharomyces	0.125	0.875	0.2658	1.9096639
Crypto-Scommu	0.529411765	0.470588235	0.9645	1.3648669
Crypto-T_indi	0.25	0.75	0.4432	0.972557
Crypto-Tramet	0.352941176	0.647058824	0.7103	1.1339349
Crypto-Umaydis	0.235294118	0.764705882	0.4017	0.912589
Crypto-Vanderwaltozyma	0.25	0.75	0.7904	1.8078792
Crypto-Yarrowia	0.375	0.625	0.8298	1.8568559
Debaryomyces-Dekkera	0.444444444	0.555555556	0.9182	1.375014
Debaryomyces-Fusarium	0.352941176	0.647058824	0.7142	1.8440622

Debaryomyces-Gibberella	0.375	0.625	0.7937	1.846125
Debaryomyces-Kluyveromyces	0.125	0.875	0.3012	1.046685
Debaryomyces-Lecanicillium	0.352941176	0.647058824	0.7167	1.957746
Debaryomyces-Metarhizium	0.352941176	0.647058824	0.7091	1.9256821
Debaryomyces-Micosphaerella	0.375	0.625	0.7633	1.7378536
Debaryomyces-Microsporum	0.375	0.625	0.793	1.7588904
Debaryomyces-Millerozyma	0.9375	0.0625	1	0.391155
Debaryomyces-Monilio	0.210526316	0.789473684	0.4616	1.9690314
Debaryomyces-mviol_mt	0.2	0.8	0.5073	1.847784
Debaryomyces-Nakaseomyces	0.125	0.875	0.3048	1.1992615
Debaryomyces-Ogataea	0.470588235	0.529411765	0.9062	1.491475
Debaryomyces-P_paky	0.470588235	0.529411765	0.909	2.299624
Debaryomyces-Paracoccidioides	0.5	0.5	0.9084	1.8615077
Debaryomyces-Peltigera	0.368421053	0.631578947	0.9146	1.8116816
Debaryomyces-Penicillium	0.25	0.75	0.3675	1.7433392
Debaryomyces-Phaeosphaeria	0.375	0.625	0.9058	1.7896236
Debaryomyces-Pichia	0.5	0.5	0.9348	1.218512
Debaryomyces-Pleurotus	0.315789474	0.684210526	0.7637	1.9032264
Debaryomyces-Podospora	0.222222222	0.777777778	0.4468	1.7986635
Debaryomyces-Rhizophydium	0.25	0.75	0.3639	2.721165
Debaryomyces-Schizosaccharomyces	0.125	0.875	0.2561	2.076859
Debaryomyces-Scommu	0.470588235	0.529411765	0.9089	2.025784
Debaryomyces-T_indi	0.5	0.5	0.8829	1.8309039
Debaryomyces-Tramet	0.352941176	0.647058824	0.7152	1.794852
Debaryomyces-Umaydis	0.352941176	0.647058824	0.7269	1.7709359
Debaryomyces-Vanderwaltozyma	0.25	0.75	0.8015	1.1247463
Debaryomyces-Yarrowia	0.5	0.5	0.9313	1.518853
Dekkera-Fusarium	0.222222222	0.777777778	0.4017	1.6522462
Dekkera-Gibberella	0.222222222	0.777777778	0.4733	1.654309
Dekkera-Kluyveromyces	0.222222222	0.777777778	0.7808	0.854869
Dekkera-Lecanicillium	0.222222222	0.777777778	0.4042	1.76593
Dekkera-Metarhizium	0.222222222	0.777777778	0.4048	1.7338661
Dekkera-Micosphaerella	0.444444444	0.555555556	0.9548	1.5460376
Dekkera-Microsporum	0.222222222	0.777777778	0.5497	1.5670744
Dekkera-Millerozyma	0.611111111	0.388888889	0.9956	1.390159
Dekkera-Monilio	0.315789474	0.684210526	0.77	1.7772154
Dekkera-mviol_mt	0.4	0.6	0.9146	1.655968
Dekkera-Nakaseomyces	0.055555556	0.944444444	0	1.0074455
Dekkera-Ogataea	0.555555556	0.444444444	0.9753	0.631981
Dekkera-P_paky	0.333333333	0.666666667	0.7081	2.107808
Dekkera-Paracoccidioides	0.222222222	0.777777778	0.4619	1.6696917
Dekkera-Peltigera	0.421052632	0.578947368	0.9032	1.6198656
Dekkera-Penicillium	0.222222222	0.777777778	0.4371	1.5515232
Dekkera-Phaeosphaeria	0.388888889	0.611111111	0.9854	1.5978076

Dekkera-Pichia	0.388888889	0.611111111	0.9366	0.582426
Dekkera-Pleurotus	0.368421053	0.631578947	0.9089	1.7114104
Dekkera-Podospora	0.222222222	0.777777778	0.4467	1.6068475
Dekkera-Rhizophydium	0.055555556	0.944444444	0	2.529349
Dekkera-Schizosaccharomyces	0.111111111	0.888888889	0.308	1.885043
Dekkera-Scommu	0.444444444	0.555555556	0.9039	1.833968
Dekkera-T_indi	0.388888889	0.611111111	0.8956	1.6390879
Dekkera-Tramet	0.388888889	0.611111111	0.8683	1.603036
Dekkera-Umaydis	0.333333333	0.666666667	0.7121	1.5791199
Dekkera-Vanderwaltozyma	0.111111111	0.888888889	0.4003	0.9329303
Dekkera-Yarrowia	0.388888889	0.611111111	0.9335	1.327037
Fusarium-Gibberella	0.882352941	0.117647059	1	0.0303382
Fusarium-Kluyveromyces	0.117647059	0.882352941	0.3284	1.3239172
Fusarium-Lecanicillium	1	0	1	0.2543268
Fusarium-Metarhizium	1	0	1	0.2222629
Fusarium-Micosphaerella	0.588235294	0.411764706	0.9912	0.488392
Fusarium-Microsporum	0.470588235	0.529411765	0.9568	0.5759586
Fusarium-Millerozyma	0.352941176	0.647058824	0.7189	1.8592072
Fusarium-Monilio	0.315789474	0.684210526	0.7463	1.3959356
Fusarium-mviol_mt	0.2	0.8	0.4655	1.2746882
Fusarium-Nakaseomyces	0.294117647	0.705882353	0.9438	1.4764937
Fusarium-Ogataea	0.411764706	0.588235294	0.8498	1.7687072
Fusarium-P_paky	0.352941176	0.647058824	0.6736	1.7265282
Fusarium-Paracoccidioides	0.647058824	0.352941176	0.9971	0.6785759
Fusarium-Peltigera	0.736842105	0.263157895	0.9999	0.56222
Fusarium-Penicillium	0.588235294	0.411764706	0.9801	0.5604074
Fusarium-Phaeosphaeria	0.235294118	0.764705882	0.6636	0.540162
Fusarium-Pichia	0.529411765	0.470588235	0.9866	1.4957442
Fusarium-Pleurotus	0.210526316	0.789473684	0.4354	1.3301306
Fusarium-Podospora	0.722222222	0.277777778	0.9994	0.3746823
Fusarium-Rhizophydium	0.411764706	0.588235294	0.8733	2.1480692
Fusarium-Schizosaccharomyces	0.117647059	0.882352941	0.2821	1.5037632
Fusarium-Scommu	0.470588235	0.529411765	0.8824	1.4526882
Fusarium-T_indi	0.705882353	0.294117647	0.9978	1.2578081
Fusarium-Tramet	0.411764706	0.588235294	0.8528	1.2217562
Fusarium-Umaydis	0.470588235	0.529411765	0.883	1.1978401
Fusarium-Vanderwaltozyma	0.117647059	0.882352941	0.3697	1.4019785
Fusarium-Yarrowia	0.647058824	0.352941176	0.997	1.4509552
Gibberella-Kluyveromyces	0.133333333	0.866666667	0.4775	1.32598
Gibberella-Lecanicillium	0.882352941	0.117647059	1	0.2563896
Gibberella-Metarhizium	0.882352941	0.117647059	0.9999	0.2243257
Gibberella-Micosphaerella	0.666666667	0.333333333	0.9825	0.4904548
Gibberella-Microsporum	0.533333333	0.466666667	0.9336	0.5780214
Gibberella-Millerozyma	0.375	0.625	0.789	1.86127
Gibberella-Monilio	0.210526316	0.789473684	0.5148	1.3979984
Gibberella-mviol_mt	0.3	0.7	0.8228	1.276751

Gibberella-Nakaseomyces	0.266666667	0.733333333	0.8307	1.4785565
Gibberella-Ogataea	0.411764706	0.588235294	0.8996	1.77077
Gibberella-P_paky	0.352941176	0.647058824	0.752	1.728591
Gibberella-Paracoccidioides	0.666666667	0.333333333	0.9871	0.6806387
Gibberella-Peltigera	0.631578947	0.368421053	0.9997	0.5642828
Gibberella-Penicillium	0.5625	0.4375	0.9851	0.5624702
Gibberella-Phaeosphaeria	0.266666667	0.733333333	0.5983	0.5422248
Gibberella-Pichia	0.666666667	0.333333333	0.9856	1.497807
Gibberella-Pleurotus	0.210526316	0.789473684	0.5004	1.3321934
Gibberella-Podospora	0.555555556	0.444444444	0.9968	0.3767451
Gibberella-Rhizophydium	0.4375	0.5625	0.9234	2.150132
Gibberella-Schizosaccharomyces	0.266666667	0.733333333	0.8655	1.505826
Gibberella-Scommu	0.352941176	0.647058824	0.7515	1.454751
Gibberella-T_indi	0.5	0.5	0.9476	1.2598709
Gibberella-Tramet	0.529411765	0.470588235	0.9754	1.223819
Gibberella-Umaydis	0.352941176	0.647058824	0.7552	1.1999029
Gibberella-Vanderwaltozyma	0.133333333	0.866666667	0.3221	1.4040413
Gibberella-Yarrowia	0.8	0.2	0.9984	1.453018
Kluyveromyces-Lecanicillium	0.117647059	0.882352941	0.3248	1.437601
Kluyveromyces-Metarhizium	0.117647059	0.882352941	0.3256	1.4055371
Kluyveromyces-Micosphaerella	0.214285714	0.785714286	0.8506	1.2177086
Kluyveromyces-Microsporum	0.066666667	0.933333333	0	1.2387454
Kluyveromyces-Millerozyma	0.25	0.75	0.7409	1.06183
Kluyveromyces-Monilio	0.105263158	0.894736842	0.2919	1.4488864
Kluyveromyces-mviol_mt	0.25	0.75	0.9289	1.327639
Kluyveromyces-Nakaseomyces	0.6	0.4	0.8294	0.4270685
Kluyveromyces-Ogataea	0.117647059	0.882352941	0.32	0.97133
Kluyveromyces-P_paky	0.352941176	0.647058824	0.9641	1.779479
Kluyveromyces-Paracoccidioides	0.133333333	0.866666667	0.3666	1.3413627
Kluyveromyces-Peltigera	0.105263158	0.894736842	0.3808	1.2915366
Kluyveromyces-Penicillium	0.125	0.875	0.3088	1.2231942
Kluyveromyces-Phaeosphaeria	0.4	0.6	0.9554	1.2694786
Kluyveromyces-Pichia	0.357142857	0.642857143	0.9833	0.698367
Kluyveromyces-Pleurotus	0.105263158	0.894736842	0.3025	1.3830814
Kluyveromyces-Podospora	0.055555556	0.944444444	0	1.2785185
Kluyveromyces-Rhizophydium	0.125	0.875	0.2981	2.20102
Kluyveromyces-Schizosaccharomyces	0.2	0.8	0.1009	1.556714
Kluyveromyces-Scommu	0.117647059	0.882352941	0.3305	1.505639
Kluyveromyces-T_indi	0.1875	0.8125	0.7067	1.3107589
Kluyveromyces-Tramet	0.058823529	0.941176471	0	1.274707
Kluyveromyces-Umaydis	0.294117647	0.705882353	0.9443	1.2507909
Kluyveromyces-Vanderwaltozyma	0.6	0.4	0.8979	0.3525533
Kluyveromyces-Yarrowia	0.214285714	0.785714286	0.8559	0.998708

Lecanicillium-Metarhizium	1	0	1	0.2809539
Lecanicillium-Micosphaerella	0.588235294	0.411764706	0.9921	0.6020758
Lecanicillium-Microsporum	0.470588235	0.529411765	0.9579	0.6896424
Lecanicillium-Millerozyma	0.352941176	0.647058824	0.7144	1.972891
Lecanicillium-Monilio	0.315789474	0.684210526	0.7454	1.5096194
Lecanicillium-mviol_mt	0.2	0.8	0.465	1.388372
Lecanicillium-Nakaseomyces	0.294117647	0.705882353	0.9427	1.5901775
Lecanicillium-Ogataea	0.411764706	0.588235294	0.847	1.882391
Lecanicillium-P_paky	0.352941176	0.647058824	0.6787	1.840212
Lecanicillium-Paracoccidioides	0.647058824	0.352941176	0.9975	0.7922597
Lecanicillium-Peltigera	0.736842105	0.263157895	0.9998	0.6759038
Lecanicillium-Penicillium	0.588235294	0.411764706	0.98	0.6740912
Lecanicillium-Phaeosphaeria	0.235294118	0.764705882	0.6582	0.6538458
Lecanicillium-Pichia	0.529411765	0.470588235	0.9822	1.609428
Lecanicillium-Pleurotus	0.210526316	0.789473684	0.4401	1.4438144
Lecanicillium-Podospora	0.722222222	0.277777778	0.9995	0.4883661
Lecanicillium-Rhizophydium	0.411764706	0.588235294	0.8771	2.261753
Lecanicillium-Schizosaccharomyces	0.117647059	0.882352941	0.2857	1.617447
Lecanicillium-Scommu	0.470588235	0.529411765	0.8833	1.566372
Lecanicillium-T_indi	0.705882353	0.294117647	0.9972	1.3714919
Lecanicillium-Tramet	0.411764706	0.588235294	0.8416	1.33544
Lecanicillium-Umaydis	0.470588235	0.529411765	0.8769	1.3115239
Lecanicillium-Vanderwaltozyma	0.117647059	0.882352941	0.3731	1.5156623
Lecanicillium-Yarrowia	0.647058824	0.352941176	0.9985	1.564639
Metarhizium-Micosphaerella	0.588235294	0.411764706	0.9918	0.5700119
Metarhizium-Microsporum	0.470588235	0.529411765	0.9545	0.6575785
Metarhizium-Millerozyma	0.352941176	0.647058824	0.7136	1.9408271
Metarhizium-Monilio	0.315789474	0.684210526	0.7287	1.4775555
Metarhizium-mviol_mt	0.2	0.8	0.4702	1.3563081
Metarhizium-Nakaseomyces	0.294117647	0.705882353	0.9414	1.5581136
Metarhizium-Ogataea	0.411764706	0.588235294	0.8477	1.8503271
Metarhizium-P_paky	0.352941176	0.647058824	0.6822	1.8081481
Metarhizium-Paracoccidioides	0.647058824	0.352941176	0.9973	0.7601958
Metarhizium-Peltigera	0.736842105	0.263157895	0.9997	0.6438399
Metarhizium-Penicillium	0.588235294	0.411764706	0.9771	0.6420273
Metarhizium-Phaeosphaeria	0.235294118	0.764705882	0.6573	0.6217819
Metarhizium-Pichia	0.529411765	0.470588235	0.9818	1.5773641
Metarhizium-Pleurotus	0.210526316	0.789473684	0.4334	1.4117505
Metarhizium-Podospora	0.722222222	0.277777778	0.9997	0.4563022
Metarhizium-Rhizophydium	0.411764706	0.588235294	0.8762	2.2296891
Metarhizium-Schizosaccharomyces	0.117647059	0.882352941	0.2854	1.5853831
Metarhizium-Scommu	0.470588235	0.529411765	0.8889	1.5343081
Metarhizium-T_indi	0.705882353	0.294117647	0.9972	1.339428
Metarhizium-Tramet	0.411764706	0.588235294	0.8508	1.3033761

Metarhizium-Umaydis	0.470588235	0.529411765	0.8823	1.27946
Metarhizium-Vanderwaltozyma	0.117647059	0.882352941	0.3798	1.4835984
Metarhizium-Yarrowia	0.647058824	0.352941176	0.9978	1.5325751
Micosphaerella-Microsporum	0.4	0.6	0.729	0.46975
Micosphaerella-Millerozyma	0.4375	0.5625	0.899	1.7529986
Micosphaerella-Monilio	0.421052632	0.578947368	0.9662	1.289727
Micosphaerella-mviol_mt	0.2	0.8	0.5723	1.1684796
Micosphaerella-Nakaseomyces	0.285714286	0.714285714	0.808	1.3702851
Micosphaerella-Ogataea	0.235294118	0.764705882	0.4797	1.6624986
Micosphaerella-P_paky	0.411764706	0.588235294	0.9224	1.6203196
Micosphaerella-Paracoccidioides	0.4	0.6	0.7161	0.5723673
Micosphaerella-Peltigera	0.526315789	0.473684211	0.9946	0.399398
Micosphaerella-Penicillium	0.375	0.625	0.7675	0.4541988
Micosphaerella-Phaeosphaeria	0.428571429	0.571428571	0.8657	0.37734
Micosphaerella-Pichia	0.428571429	0.571428571	0.6795	1.3895356
Micosphaerella-Pleurotus	0.421052632	0.578947368	0.9639	1.223922
Micosphaerella-Podospora	0.222222222	0.777777778	0.5975	0.4429933
Micosphaerella-Rhizophydium	0.3125	0.6875	0.7181	2.0418606
Micosphaerella-Schizosaccharomyces	0.071428571	0.928571429	0	1.3975546
Micosphaerella-Scommu	0.470588235	0.529411765	0.9438	1.3464796
Micosphaerella-T_indi	0.5625	0.4375	0.979	1.1515995
Micosphaerella-Tramet	0.235294118	0.764705882	0.4832	1.1155476
Micosphaerella-Umaydis	0.352941176	0.647058824	0.7798	1.0916315
Micosphaerella-Vanderwaltozyma	0.285714286	0.714285714	0.7456	1.2957699
Micosphaerella-Yarrowia	0.785714286	0.214285714	0.9927	1.3447466
Microsporum-Millerozyma	0.125	0.875	0.1559	1.7740354
Microsporum-Monilio	0.315789474	0.684210526	0.8627	1.3107638
Microsporum-mviol_mt	0.1	0.9	0.2197	1.1895164
Microsporum-Nakaseomyces	0.066666667	0.933333333	0	1.3913219
Microsporum-Ogataea	0.470588235	0.529411765	0.9586	1.6835354
Microsporum-P_paky	0.117647059	0.882352941	0.1675	1.6413564
Microsporum-Paracoccidioides	0.866666667	0.133333333	0.9997	0.3766027
Microsporum-Peltigera	0.210526316	0.789473684	0.5836	0.543578
Microsporum-Penicillium	0.5	0.5	0.944	0.3376372
Microsporum-Phaeosphaeria	0.133333333	0.866666667	0.2039	0.52152
Microsporum-Pichia	0.466666667	0.533333333	0.8735	1.4105724
Microsporum-Pleurotus	0.105263158	0.894736842	0.2059	1.2449588
Microsporum-Podospora	0.277777778	0.722222222	0.8748	0.5305599
Microsporum-Rhizophydium	0.375	0.625	0.7883	2.0628974
Microsporum-Schizosaccharomyces	0.133333333	0.866666667	0.4331	1.4185914
Microsporum-Scommu	0.235294118	0.764705882	0.5274	1.3675164
Microsporum-T_indi	0.125	0.875	0.1572	1.1726363

Microsporum-Tramet	0.235294118	0.764705882	0.5257	1.1365844
Microsporum-Umaydis	0.235294118	0.764705882	0.5214	1.1126683
Microsporum-Vanderwaltozyma	0.066666667	0.933333333	0	1.3168067
Microsporum-Yarrowia	0.4	0.6	0.7212	1.3657834
Millerozyma-Monilio	0.315789474	0.684210526	0.774	1.9841764
Millerozyma-mviol_mt	0.2	0.8	0.4974	1.862929
Millerozyma-Nakaseomyces	0.125	0.875	0.3	1.2144065
Millerozyma-Ogataea	0.588235294	0.411764706	0.9806	1.50662
Millerozyma-P_paky	0.470588235	0.529411765	0.9067	2.314769
Millerozyma-Paracoccidioides	0.25	0.75	0.4021	1.8766527
Millerozyma-Peltigera	0.368421053	0.631578947	0.9137	1.8268266
Millerozyma-Penicillium	0.375	0.625	0.6726	1.7584842
Millerozyma-Phaeosphaeria	0.375	0.625	0.9057	1.8047686
Millerozyma-Pichia	0.5	0.5	0.9329	1.233657
Millerozyma-Pleurotus	0.473684211	0.526315789	0.9798	1.9183714
Millerozyma-Podospora	0.222222222	0.777777778	0.4577	1.8138085
Millerozyma-Rhizophydium	0.125	0.875	0.1016	2.73631
Millerozyma-Schizosaccharomyces	0.0625	0.9375	0	2.092004
Millerozyma-Scommu	0.411764706	0.588235294	0.881	2.040929
Millerozyma-T_indi	0.5	0.5	0.8879	1.8460489
Millerozyma-Tramet	0.294117647	0.705882353	0.6793	1.809997
Millerozyma-Umaydis	0.411764706	0.588235294	0.8737	1.7860809
Millerozyma-Vanderwaltozyma	0.25	0.75	0.803	1.1398913
Millerozyma-Yarrowia	0.5	0.5	0.9308	1.533998
Monilio-mviol_mt	0.55	0.45	0.9923	0.9059354
Monilio-Nakaseomyces	0.315789474	0.684210526	0.9777	1.6014629
Monilio-Ogataea	0.210526316	0.789473684	0.4297	1.8936764
Monilio-P_paky	0.210526316	0.789473684	0.4396	1.3577754
Monilio-Paracoccidioides	0.315789474	0.684210526	0.8059	1.4133811
Monilio-Peltigera	0.421052632	0.578947368	0.9152	1.363555
Monilio-Penicillium	0.105263158	0.894736842	0.1459	1.2952126
Monilio-Phaeosphaeria	0.210526316	0.789473684	0.7181	1.341497
Monilio-Pichia	0.315789474	0.684210526	0.8385	1.6207134
Monilio-Pleurotus	0.684210526	0.315789474	0.9983	0.368331
Monilio-Podospora	0.421052632	0.578947368	0.9111	1.3505369
Monilio-Rhizophydium	0.421052632	0.578947368	0.9344	2.0623164
Monilio-Schizosaccharomyces	0.105263158	0.894736842	0.335	1.6287324
Monilio-Scommu	0.473684211	0.526315789	0.9682	0.5889354
Monilio-T_indi	0.315789474	0.684210526	0.778	0.8890553
Monilio-Tramet	0.578947368	0.421052632	0.995	0.4904254
Monilio-Umaydis	0.421052632	0.578947368	0.922	0.8290873
Monilio-Vanderwaltozyma	0.210526316	0.789473684	0.8541	1.5269477
Monilio-Yarrowia	0.421052632	0.578947368	0.9636	1.5759244
mviol_mt-Nakaseomyces	0.1	0.9	0.3945	1.4802155
mviol_mt-Ogataea	0.2	0.8	0.4603	1.772429

mviol_mt-P_paky	0.45	0.55	0.9771	0.945058
mviol_mt-Paracoccidioides	0.1	0.9	0.1821	1.2921337
mviol_mt-Peltigera	0.2	0.8	0.4111	1.2423076
mviol_mt-Penicillium	0.1	0.9	0.1707	1.1739652
mviol_mt-Phaeosphaeria	0.3	0.7	0.9529	1.2202496
mviol_mt-Pichia	0.4	0.6	0.9666	1.499466
mviol_mt-Pleurotus	0.3	0.7	0.7117	0.8401304
mviol_mt-Podospora	0.2	0.8	0.4454	1.2292895
mviol_mt-Rhizophydium	0.2	0.8	0.5017	1.941069
mviol_mt-Schizosaccharomyces	0.2	0.8	0.7779	1.507485
mviol_mt-Scommu	0.5	0.5	0.9858	0.962688
mviol_mt-T_indi	0.35	0.65	0.9249	0.6307325
mviol_mt-Tramet	0.4	0.6	0.932	0.731756
mviol_mt-Umaydis	0.25	0.75	0.73	0.5707645
mviol_mt-Vanderwaltozyma	0.25	0.75	0.9802	1.4057003
mviol_mt-Yarrowia	0.4	0.6	0.9716	1.454677
Nakaseomyces-Ogataea	0.117647059	0.882352941	0.3266	1.1239065
Nakaseomyces-P_paky	0.235294118	0.764705882	0.7643	1.9320555
Nakaseomyces-Paracoccidioides	0.133333333	0.866666667	0.2733	1.4939392
Nakaseomyces-Peltigera	0.315789474	0.684210526	0.9781	1.4441131
Nakaseomyces-Penicillium	0.1875	0.8125	0.6988	1.3757707
Nakaseomyces-Phaeosphaeria	0.1	0.9	0	1.4220551
Nakaseomyces-Pichia	0.357142857	0.642857143	0.9602	0.8509435
Nakaseomyces-Pleurotus	0.263157895	0.736842105	0.9631	1.5356579
Nakaseomyces-Podospora	0.111111111	0.888888889	0.4563	1.431095
Nakaseomyces-Rhizophydium	0.4375	0.5625	0.9928	2.3535965
Nakaseomyces-Schizosaccharomyces	0.444444444	0.555555556	0.397	1.7092905
Nakaseomyces-Scommu	0.235294118	0.764705882	0.767	1.6582155
Nakaseomyces-T_indi	0.25	0.75	0.7453	1.4633354
Nakaseomyces-Tramet	0.235294118	0.764705882	0.7686	1.4272835
Nakaseomyces-Umaydis	0.176470588	0.823529412	0.7341	1.4033674
Nakaseomyces-Vanderwaltozyma	0.875	0.125	0.9487	0.4220192
Nakaseomyces-Yarrowia	0.214285714	0.785714286	0.768	1.1512845
Ogataea-P_paky	0.588235294	0.411764706	0.9739	2.224269
Ogataea-Paracoccidioides	0.411764706	0.588235294	0.9014	1.7861527
Ogataea-Peltigera	0.526315789	0.473684211	0.9839	1.7363266
Ogataea-Penicillium	0.705882353	0.294117647	0.9974	1.6679842
Ogataea-Phaeosphaeria	0.235294118	0.764705882	0.6609	1.7142686
Ogataea-Pichia	0.588235294	0.411764706	0.9912	0.698887
Ogataea-Pleurotus	0.473684211	0.526315789	0.9722	1.8278714
Ogataea-Podospora	0.222222222	0.777777778	0.4128	1.7233085
Ogataea-Rhizophydium	0.058823529	0.941176471	0	2.64581
Ogataea-Schizosaccharomyces	0.058823529	0.941176471	0	2.001504

Ogataea-Scommu	0.470588235	0.529411765	0.8824	1.950429
Ogataea-T_indi	0.470588235	0.529411765	0.9066	1.7555489
Ogataea-Tramet	0.411764706	0.588235294	0.8496	1.719497
Ogataea-Umaydis	0.117647059	0.882352941	0.1049	1.6955809
Ogataea-Vanderwaltozyma	0.058823529	0.941176471	0	1.0493913
Ogataea-Yarrowia	0.470588235	0.529411765	0.9429	1.443498
P_paky-Paracoccidioides	0.352941176	0.647058824	0.7563	1.7439737
P_paky-Peltigera	0.315789474	0.684210526	0.7519	1.6941476
P_paky-Penicillium	0.352941176	0.647058824	0.7154	1.6258052
P_paky-Phaeosphaeria	0.235294118	0.764705882	0.6622	1.6720896
P_paky-Pichia	0.705882353	0.294117647	0.9997	1.951306
P_paky-Pleurotus	0.421052632	0.578947368	0.9246	1.2919704
P_paky-Podospora	0.222222222	0.777777778	0.4156	1.6811295
P_paky-Rhizophydium	0.352941176	0.647058824	0.7103	2.392909
P_paky-Schizosaccharomyces	0.117647059	0.882352941	0.2818	1.959325
P_paky-Scommu	0.411764706	0.588235294	0.8535	1.414528
P_paky-T_indi	0.588235294	0.411764706	0.9795	1.0825725
P_paky-Tramet	0.235294118	0.764705882	0.3717	1.183596
P_paky-Umaydis	0.294117647	0.705882353	0.6434	1.0226045
P_paky-Vanderwaltozyma	0.117647059	0.882352941	0.374	1.8575403
P_paky-Yarrowia	0.588235294	0.411764706	0.9919	1.906517
Paracoccidioides-Peltigera	0.315789474	0.684210526	0.8044	0.6461953
Paracoccidioides-Penicillium	0.625	0.375	0.9818	0.4402545
Paracoccidioides-Phaeosphaeria	0.133333333	0.866666667	0.2072	0.6241373
Paracoccidioides-Pichia	0.6	0.4	0.9682	1.5131897
Paracoccidioides-Pleurotus	0.105263158	0.894736842	0.1639	1.3475761
Paracoccidioides-Podospora	0.444444444	0.555555556	0.9678	0.6331772
Paracoccidioides-Rhizophydium	0.5625	0.4375	0.9659	2.1655147
Paracoccidioides-Schizosaccharomyces	0.133333333	0.866666667	0.3209	1.5212087
Paracoccidioides-Scommu	0.235294118	0.764705882	0.4363	1.4701337
Paracoccidioides-T_indi	0.375	0.625	0.7147	1.2752536
Paracoccidioides-Tramet	0.235294118	0.764705882	0.4408	1.2392017
Paracoccidioides-Umaydis	0.352941176	0.647058824	0.7532	1.2152856
Paracoccidioides-Vanderwaltozyma	0.133333333	0.866666667	0.3222	1.419424
Paracoccidioides-Yarrowia	0.533333333	0.466666667	0.9152	1.4684007
Peltigera-Penicillium	0.421052632	0.578947368	0.9407	0.5280268
Peltigera-Phaeosphaeria	0.210526316	0.789473684	0.7178	0.379104
Peltigera-Pichia	0.315789474	0.684210526	0.8339	1.4633636
Peltigera-Pleurotus	0.315789474	0.684210526	0.7326	1.29775
Peltigera-Podospora	0.526315789	0.473684211	0.9911	0.5168213
Peltigera-Rhizophydium	0.263157895	0.736842105	0.7454	2.1156886
Peltigera-Schizosaccharomyces	0.105263158	0.894736842	0.3301	1.4713826
Peltigera-Scommu	0.421052632	0.578947368	0.9206	1.4203076

Peltigera-T_indi	0.421052632	0.578947368	0.9376	1.2254275
Peltigera-Tramet	0.421052632	0.578947368	0.922	1.1893756
Peltigera-Umaydis	0.368421053	0.631578947	0.8953	1.1654595
Peltigera-Vanderwaltozyma	0.210526316	0.789473684	0.8568	1.3695979
Peltigera-Yarrowia	0.421052632	0.578947368	0.9652	1.4185746
Penicillium-Phaeosphaeria	0.125	0.875	0.2312	0.5059688
Penicillium-Pichia	0.5625	0.4375	0.9786	1.3950212
Penicillium-Pleurotus	0.210526316	0.789473684	0.4706	1.2294076
Penicillium-Podospora	0.333333333	0.666666667	0.7666	0.5150087
Penicillium-Rhizophydium	0.3125	0.6875	0.6364	2.0473462
Penicillium-Schizosaccharomyces	0.0625	0.9375	0	1.4030402
Penicillium-Scommu	0.352941176	0.647058824	0.7137	1.3519652
Penicillium-T_indi	0.4375	0.5625	0.8493	1.1570851
Penicillium-Tramet	0.352941176	0.647058824	0.7134	1.1210332
Penicillium-Umaydis	0.352941176	0.647058824	0.7132	1.0971171
Penicillium-Vanderwaltozyma	0.125	0.875	0.3529	1.3012555
Penicillium-Yarrowia	0.3125	0.6875	0.7253	1.3502322
Phaeosphaeria-Pichia	0.285714286	0.714285714	0.5609	1.4413056
Phaeosphaeria-Pleurotus	0.210526316	0.789473684	0.7171	1.275692
Phaeosphaeria-Podospora	0.222222222	0.777777778	0.6933	0.4947633
Phaeosphaeria-Rhizophydium	0.0625	0.9375	0	2.0936306
Phaeosphaeria-Schizosaccharomyces	0.1	0.9	0	1.4493246
Phaeosphaeria-Scommu	0.352941176	0.647058824	0.9221	1.3982496
Phaeosphaeria-T_indi	0.25	0.75	0.6423	1.2033695
Phaeosphaeria-Tramet	0.235294118	0.764705882	0.6556	1.1673176
Phaeosphaeria-Umaydis	0.235294118	0.764705882	0.653	1.1434015
Phaeosphaeria-Vanderwaltozyma	0.4	0.6	0.8932	1.3475399
Phaeosphaeria-Yarrowia	0.285714286	0.714285714	0.5528	1.3965166
Pichia-Pleurotus	0.315789474	0.684210526	0.8397	1.5549084
Pichia-Podospora	0.222222222	0.777777778	0.608	1.4503455
Pichia-Rhizophydium	0.125	0.875	0.1316	2.372847
Pichia-Schizosaccharomyces	0.142857143	0.857142857	0.4041	1.728541
Pichia-Scommu	0.470588235	0.529411765	0.9463	1.677466
Pichia-T_indi	0.5625	0.4375	0.9762	1.4825859
Pichia-Tramet	0.235294118	0.764705882	0.4848	1.446534
Pichia-Umaydis	0.470588235	0.529411765	0.9401	1.4226179
Pichia-Vanderwaltozyma	0.214285714	0.785714286	0.7095	0.7764283
Pichia-Yarrowia	0.857142857	0.142857143	0.9975	1.170535
Pleurotus-Podospora	0.368421053	0.631578947	0.8802	1.2847319
Pleurotus-Rhizophydium	0.210526316	0.789473684	0.4618	1.9965114
Pleurotus-Schizosaccharomyces	0.105263158	0.894736842	0.3385	1.5629274
Pleurotus-Scommu	0.368421053	0.631578947	0.8944	0.5231304
Pleurotus-T_indi	0.210526316	0.789473684	0.4732	0.8232503

Pleurotus-Tramet	0.526315789	0.473684211	0.9833	0.4246204
Pleurotus-Umaydis	0.105263158	0.894736842	0.1365	0.7632823
Pleurotus-Vanderwaltozyma	0.105263158	0.894736842	0.4298	1.4611427
Pleurotus-Yarrowia	0.210526316	0.789473684	0.5479	1.5101194
Podospora-Rhizophydium	0.222222222	0.777777778	0.4461	2.1026705
Podospora-Schizosaccharomyces	0.111111111	0.888888889	0.308	1.4583645
Podospora-Scommu	0.222222222	0.777777778	0.419	1.4072895
Podospora-T_indi	0.555555556	0.444444444	0.9888	1.2124094
Podospora-Tramet	0.555555556	0.444444444	0.9848	1.1763575
Podospora-Umaydis	0.333333333	0.666666667	0.7365	1.1524414
Podospora-Vanderwaltozyma	0.055555556	0.944444444	0	1.3565798
Podospora-Yarrowia	0.222222222	0.777777778	0.5997	1.4055565
Rhizophydium-Schizosaccharomyces	0.125	0.875	0.2623	2.380866
Rhizophydium-Scommu	0.352941176	0.647058824	0.7069	2.119069
Rhizophydium-T_indi	0.25	0.75	0.3732	1.9241889
Rhizophydium-Tramet	0.235294118	0.764705882	0.4	1.888137
Rhizophydium-Umaydis	0.235294118	0.764705882	0.4057	1.8642209
Rhizophydium-Vanderwaltozyma	0.25	0.75	0.8002	2.2790813
Rhizophydium-Yarrowia	0.375	0.625	0.7576	2.328058
Schizosaccharomyces-Scommu	0.058823529	0.941176471	0	1.685485
Schizosaccharomyces-T_indi	0.25	0.75	0.6809	1.4906049
Schizosaccharomyces-Tramet	0.235294118	0.764705882	0.7222	1.454553
Schizosaccharomyces-Umaydis	0.117647059	0.882352941	0.2804	1.4306369
Schizosaccharomyces-Vanderwaltozyma	0.222222222	0.777777778	0.1241	1.6347753
Schizosaccharomyces-Yarrowia	0.142857143	0.857142857	0.4031	1.683752
Scommu-T_indi	0.352941176	0.647058824	0.7138	0.9458079
Scommu-Tramet	0.411764706	0.588235294	0.8457	0.547178
Scommu-Umaydis	0.235294118	0.764705882	0.3658	0.8858399
Scommu-Vanderwaltozyma	0.117647059	0.882352941	0.3857	1.5837003
Scommu-Yarrowia	0.470588235	0.529411765	0.945	1.632677
T_indi-Tramet	0.352941176	0.647058824	0.7105	0.7148759
T_indi-Umaydis	0.705882353	0.294117647	0.9975	0.343554
T_indi-Vanderwaltozyma	0.1875	0.8125	0.768	1.3888202
T_indi-Yarrowia	0.5625	0.4375	0.9762	1.4377969
Tramet-Umaydis	0.235294118	0.764705882	0.3722	0.6549079
Tramet-Vanderwaltozyma	0.058823529	0.941176471	0	1.3527683
Tramet-Yarrowia	0.235294118	0.764705882	0.4865	1.401745
Umaydis-Vanderwaltozyma	0.294117647	0.705882353	0.9624	1.3288522
Umaydis-Yarrowia	0.294117647	0.705882353	0.7536	1.3778289
Vanderwaltozyma-Yarrowia	0.214285714	0.785714286	0.7083	1.0767693

NOTE.-

[a] GOC (gene order conservation) values were estimated as the number of contiguous ortholog pairs that are in common between compared genomes,

normalized by the number of shared orthologs (see Rocha 2006 for more details).
[b] GOL (gene order loss) = 1-GOC
[c] Phylogenetic (patristic) distance

Supplementary Table 2

Correlation of bsGOL with different variables including branch length, GOL rate, number of tRNAs, number of intronic ORFs, and number of introns present in the analyzed dikarya genomes

bsGOL	variables	Pearson's coefficient	p-value
GOL_branch_specific	Branch_Length	0.394687636	0.014197918
GOL_branch_specific	GOL_rate	-0.415040465	0.009568107
GOL_branch_specific	tRNAs	-0.13287618	0.426452186
GOL_branch_specific	IntronicORFs	-0.055378586	0.741228926
GOL_branch_specific	Introns	0.147390348	0.377203409

Supplementary Table 3
List of the species analyzed in the basal fungi dataset, accessions and references

Species	Taxonomy[a]	GenBank accession	Reference[b]
Allomyces_macrogynus	Bl	NC_001715	Paquin and Lang 1996
Blastocladiella_emersonii	Bl	NC_011360	Humberto et al. 2008
Gigaspora_margarita	G	NC_016684	Pelin et al. 2012
Glomus_intraradices	G	NC_012056	Lee and Young 2009
Harpochytrium_sp_JEL105	M	NC_004623	Bullerwell and Lang 2003
Hyaloraphidium_curvatum	M	NC_003048	Forget et al. 2002
Monoblepharella_sp_JEL15	M	NC_004624	Bullerwell and Lang 2003
Rozella_allomycis	Cr	NC_021611	James et al. 2013
Rhizophydium_sp_136	Ch	NC_003053	Forget et al. 2002

[a]Taxonomy: Bl = Blastocladiomycota; G = Glomeromycota; M = Monoblepharidomycota; Cr = Cryptomycota; Ch = Chytridiomycota.
[b]References not included in the Literature Cited: Humberto et al. 2008. Gene 424:33-39; Pelin et al. 2012. New Phytologist 194:836-845; Lee and Young 2009. New Phytologist 183:200-2011; Bullerwell and Lang 2003. NAR 31:1614-1623; James et al. 2013. Curr. Biol. 23:1548-1553; Forget et al. 2002.

Supplementary Table 4

Pairwise gene order conservation and loss between all pairs of basal fungi

**Species**	**GOC[a]**	**GOL[b]**	**p-value**	**Distance[c]**
Allomyces-B. emersonii	1	0	1	0.212631
Allomyces-Gigaspora	0.235294118	0.764705882	0.38144	0.8515046
Allomyces-Glomus	0.411764706	0.588235294	0.89646	0.7709755
Allomyces-Harpochytrium	0.058823529	0.941176471	0	0.8787302
Allomyces-H. curvatum	0.058823529	0.941176471	0	1.025922
Allomyces-Monoblepharella	0.058823529	0.941176471	0	0.8968586
Allomyces-R. allomycis	0.176470588	0.823529412	0.73678	2.001672
Allomyces-Rhizophydium	0.235294118	0.764705882	0.40604	1.006383
B. emersonii-Gigaspora	0.235294118	0.764705882	0.38266	0.8249729
B. emersonii-Glomus	0.411764706	0.588235294	0.89808	0.7444437
B. emersonii-Harpochytrium	0.058823529	0.941176471	0	0.8521985
B. emersoni-H. curvatum	0.058823529	0.941176471	0	0.9993904
B. emersonii-Monoblepharella	0.058823529	0.941176471	0	0.8703269
B. emersonii-R. allomycis	0.176470588	0.823529412	0.73745	1.97514
B. emersonii-Rhizophydium	0.235294118	0.764705882	0.40462	0.9798512
Gigaspora-Glomus	0.470588235	0.529411765	0.89635	0.4795584
Gigaspora-Harpochytrium	0.117647059	0.882352941	0.08908	1.034834
Gigaspora-H. curvatum	0.352941176	0.647058824	0.65545	1.182026
Gigaspora-Monoblepharella	0.117647059	0.882352941	0.08865	1.052963
Gigaspora-R. allomycis	0.058823529	0.941176471	0	2.157776
Gigaspora-Rhizophydium	0.117647059	0.882352941	0.08896	1.162487
Glomus-Harpochytrium	0.117647059	0.882352941	0.11696	0.9543051
Glomus-H. curvatum	0.117647059	0.882352941	0.11726	1.101497
Glomus-Monoblepharella	0.117647059	0.882352941	0.11733	0.9724335
Glomus-R. allomycis	0.058823529	0.941176471	0	2.077247
Glomus-Rhizophydium	0.235294118	0.764705882	0.40511	1.081958
Harpochytrium-H. curvatum	0.125	0.875	0.10324	0.4911955
Harpochytrium-Monoblepharella	1	0	0.999999	0.1801027
Harpochytrium-R. allomycis	0.0625	0.9375	0	1.923161
Harpochytrium-Rhizophydium	0.125	0.875	0.10238	0.9278721
H. curvatum-Monoblepharella	0.0625	0.9375	0	0.5093239
H. curvatum-R. allomycis	0.1875	0.8125	0.71137	2.070353
H. curvatum-Rhizophydium	0.375	0.625	0.67822	1.075064

Monoblepharella-R. allomycis	0.125	0.875	0.29952	1.941289
Monoblepharella-Rhizophydium	0.5	0.5	0.88639	0.9460004
R.allomycis-Rhizophydium	0.25	0.75	0.74198	1.734984

NOTE.-

[a] GOC (gene order conservation) values were estimated as the number of contiguous ortholog pairs that are in common between compared genomes, normalized by the number of shared orthologs (see Rocha 2006 for more details).

[b] GOL (gene order loss) = 1-GOC

[c] Phylogenetic (patristic) distance

Supplementary Table 5

Branch-specific GOL, NRPS rates, summary of the number of different genomic elements per basal fungi species

Taxon	bsGOL[a]	Branch lengths[b]	GOLrate[c]	Normalized GOL rate[d]	NPRS rates[e]	tRNAs[f]	T Es[g]	ORFs[h]	ign_repeats[i]	Genome Size[k]
Allomyces macrogynus	0.00	0.12	0.0	0.00	0.0009	25	6	14	83	57473
Blastocladiella emersonii	0.00	0.093	0.0	0.00	0.0007	26	1	2	102	36503
Gigaspora margarita	0.28	0.28	1.01	1.77	0.0013	24	0	5	570	96998
Glomus iradicens	0.25	0.2	1.24	2.19	0.0009	26	6	6	119	70606
Harpochytrium sp. JEL105	0.04	0.081	0.51	0.91	0.0005	8	0	0	10	24169
Hyalosporidium curvatum	0.40	0.319	1.24	2.19	0.0011	7	0	4	21	29593
Monoblepharella sp. JEL15	0.00	0.099	0.0	0.00	0.0006	9	5	12	132	60432
Rozella allomycis	0.43	1.37	0.31	0.55	0.0019	4	0	1	158	12055
Rhizophydium sp. 136	0.29	0.37	0.79	1.39	0.0005	7	17	23	312	68834

NOTE.-

[a] bsGOL values were estimated by minimizing the following expression: $L = \sum(\sum b_{i,j}x_j - GOL_i)^2$, where $b_{i,j}$ is a Boolean variable that specifies the branches that are relevant for the estimation of a particular branch-specific GOL (i.e. 0 if it is not relevant and 1 if it is), x_j is obtained by minimizing L and is the actual branch-specific GOL value, and GOL_i are the estimated values from the pairwise comparisons, in other words, $GOL_i = 1 - GOC_i$ (see Fischer et al. 2006 for more details).

[b] These branch lengths were obtained by maximum likelihood phylogenetic reconstruction with RaxML (Stamatakis 2006)

[c] bsGOL normalized by the branch length

[d] bsGOL rates normalized relative to the mean GOL rate value
[e] Rates obtained with r8s with the non-parametric method minimizing local transformations (NPRS) and optimization via Powell's method (Sanderson 2003)
[f] Number of tRNAs (GenBank)
[g] Number of Transposable Elements (Homing Endonucleases) (GenBank)
[h] Number of ORFs (GenBank)
[i] Number of intergenic repeats detected with mreps (Kolpakov et al. 2003)
[j] Genome size in bp (GenBank)
