# Supplementary material for: High Variability of Mitochondrial Gene Order among Fungi
Source: Genome Biol Evol. 2014 Feb 6;6(2):451–65. doi: 10.1093/gbe/evu028 (PMC3942027; doi:10.1093/gbe/evu028)
Supplement: Supplementary Data [file supp_evu028_Supplementary_Figure_Legends.pdf]

Supplementary Figure Legends

Fig. 1.- Maximum likelihood phylogeny of our sampled taxa including a total of 9 species. The gene tree was inferred The gene tree was inferred from a concatenated alignment of 14 single-copy, orthologous genes (*atp6, atp8, atp9, nad1-nad6, nad4L,cob, cox1-cox3*). RAxML v.7.2.6 (Stamatakis 2006) was used assuming the LG substitution matrix and default parameters. On the right side of each taxon name is a series of colored boxes representing the mt gene order according to GenBank annotation. Bootstrap support appears next to each node. Branch-specific GOL values are shown next to each species name and they are estimated by minimizing the following expression: $L = \sum(\sum b_{i,j} x_j - GOL_i)^2$, where $b_{i,j}$ is a Boolean variable that specifies the branches that are relevant for the estimation of a particular branch-specific GOL (i.e. 0 if it is not relevant and 1 if it is), x_j is obtained by minimizing L and is the actual branch-specific GOL value, and GOL_i are the estimated values from the pairwise comparisons, in other words, $GOL_i = 1- GOC_i$ (see Fischer et al. 2006 for more details). This figure was made using the ETE python environment for tree exploration (Huerta-Cepas et al. 2010)

Fig. 2.- GOC between pairs of genomes as a function of their phylogenetic (patristic) distance. Distances were estimated using the estimated branch lengths in figure 3, listed in table 2. Models are fitted by non-linear regression. Model 0: $GOC = 2/1+e^{\alpha t}$. Model 1: $GOC = 1 - \sqrt{\alpha t}$. Model 2: $1/GOC = \alpha t +1$. Model 3: $GOC = p^t$, where parameter α is adjusted by regression and t is the patristic distance between the two compared taxa.

Fig. 3.- Pearson's correlation between bsGOC values and branch lengths (R = 0.7, p-value = 0.004).
